# Jurassic Park approached: a coccid from Kimmeridgian cheirolepidiacean Aintourine Lebanese amber

**DOI:** 10.1093/nsr/nwae200

**Published:** 2024-06-11

**Authors:** Peter Vršanský, Hemen Sendi, Júlia Kotulová, Jacek Szwedo, Martina Havelcová, Helena Palková, Lucia Vršanská, Jakub Sakala, L'ubica Puškelová, Marián Golej, Adrian Biroň, Daniel Peyrot, Donald Quicke, Didier Néraudeau, Pavel Uher, Sibelle Maksoud, Dany Azar

**Affiliations:** Earth Science Institute v.v.i., Slovak Academy of Sciences; Dúbravská cesta 9, 840 05 Bratislava, and Ďumbierska 1, 97401 Banská Bystrica, Slovakia; Institute of Zoology v.v.i., Slovak Academy of Sciences; Dúbravska cesta 9, 84506 Bratislava, Slovakia; Slovak Academy of Sciences, Institute of Physics v.v.i., Research Center of Quantum Informatics; Dúbravská cesta 9, Bratislava 84511, Slovakia; Institute of Zoology v.v.i., Slovak Academy of Sciences; Dúbravska cesta 9, 84506 Bratislava, Slovakia; Earth Science Institute v.v.i., Slovak Academy of Sciences; Dúbravská cesta 9, 840 05 Bratislava, and Ďumbierska 1, 97401 Banská Bystrica, Slovakia; University of Gdańsk, Department of Invertebrate Zoology and Parasitology, Laboratory of Evolutionary Entomology and Museum of Amber Inclusions; Museum of Amber Inclusions; PL-80308 Gdansk, Poland; Institute of Rock Structure and Mechanics of the Czech Academy of Sciences, 18209 Praha 8, Czech Republic; Slovak Academy of Sciences, Institute of Inorganic Chemistry v.v.i.; Dúbravská cesta 9, Bratislava 84536, Slovakia; Institute of Zoology v.v.i., Slovak Academy of Sciences; Dúbravska cesta 9, 84506 Bratislava, Slovakia; AMBA projekty; Tichá 4, 81102 Bratislava, Slovakia; Institute of Geology and Palaeontology, Faculty of Science, Charles University; Albertov 6, 12843 Prague 2, Czech Republic; Earth Science Institute v.v.i., Slovak Academy of Sciences; Dúbravská cesta 9, 840 05 Bratislava, and Ďumbierska 1, 97401 Banská Bystrica, Slovakia; Earth Science Institute v.v.i., Slovak Academy of Sciences; Dúbravská cesta 9, 840 05 Bratislava, and Ďumbierska 1, 97401 Banská Bystrica, Slovakia; Earth Science Institute v.v.i., Slovak Academy of Sciences; Dúbravská cesta 9, 840 05 Bratislava, and Ďumbierska 1, 97401 Banská Bystrica, Slovakia; MGPalaeo Pty. Ltd., Malaga WA 6090, Australia; School of Biological Science; The University of Western Australia, Crawley WA 6009, Australia; Integrative Insect Ecology Research Unit, Department of Biology, Faculty of Science, Chulalongkorn University, Pathumwan, BKK 10330, Thailand; University of Rennes; UMR 6118, Géosciences Rennes, Campus de Beaulieu, Avenue du Général Leclerc, 35042 Rennes, France; Department of Mineralogy, Petrology and Economic Geology, Faculty of Natural Science, Comenius University; Ilkovičova 6, 84215 Bratislava, Slovakia; Lebanese University, Faculty of Sciences II, Department of Natural Sciences, Jdeideh, Matn, Lebanon; State Key Laboratory of Palaeobiology and Petroleum Stratigraphy, Nanjing Institute of Geology and Palaeontology, Chinese Academy of Sciences, Nanjing 210008, China; Lebanese University, Faculty of Sciences II, Department of Natural Sciences, Jdeideh, Matn, Lebanon; State Key Laboratory of Palaeobiology and Petroleum Stratigraphy, Nanjing Institute of Geology and Palaeontology, Chinese Academy of Sciences, Nanjing 210008, China

**Keywords:** fossil insect, Jurassic amber, Lebanon, evolution, new family

## Abstract

With the exception of a fly and a mite from the Triassic of Italy, all Mesozoic amber arthropods are from the Cretaceous. Late Jurassic Lebanese amber from Aintourine revealed a completely preserved adult coccid male (wing length 0.8 mm), *Jankotejacoccus libanogloria* gen. et sp. n., the earliest record of a plant sucking scale insect. Associated plant material included the cheirolepidiaceans *Protopodocarpoxylon, Brachyphyllum* and *Classostrobus*, plus *Classopoli*s pollen, suggesting a forested temporary swamp habitat with ferns, tree ferns, water ferns, tall araucarian and ginkgoacean trees and shrubs. (Sub)tropic lateritic soil with vegetation debris underwent incomplete microbial decomposition in an anoxic water environment of peat swamp development. Strata-associated marine organisms support the Kimmeridgian age revealed by zircons. The discovery opens a new field of research in Jurassic amber fossils.

## INTRODUCTION

**Figure 1.**
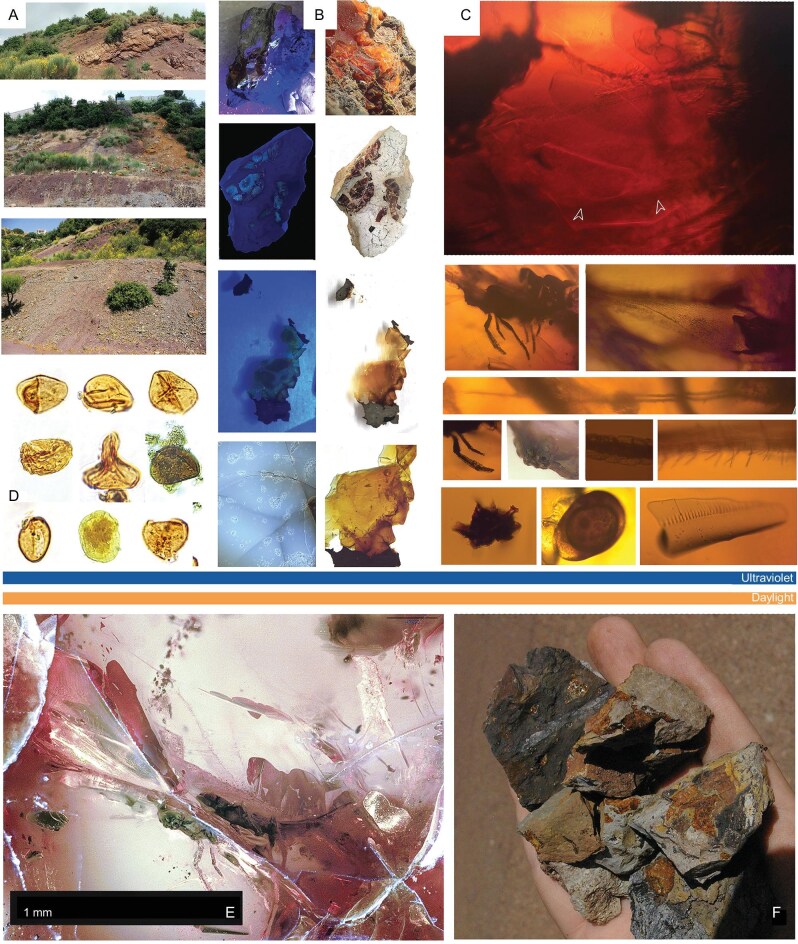
First Jurassic insect from amber (Kimmeridgian Aintourine, Lebanon). (A) Locality and its surrounding (see MM). (B) Rough and prepared samples in UV and white light—from the same rock piece as the holotype. (C and E) Details of the insect inclusion *Jankotejacoccus libanogloria* Szwedo, Azar et Sendi, sp. n., holotype, SNM Z 40023A and syninclusions. (D) Pollen (SP-33219–1.1–4): upper row, spores of a tree fern affiliated to Cyatheaceae/Dicksoniaceae (2× *Cyathidites* spp., *Deltoidospora* sp.); middle row, spores of Schizaeales (*Ischyosporites* sp.), Matoniaceae (*Dictyophyllidites* sp.), Marsileaceae (*Peromonolites allenensis*); bottom row, pollen of Ginkgoales/Cycadales (*Cycadopites* sp.), Araucariaceae (*Araucariacites australis*), Cheirolepidiaceae (*Classopollis* sp.). (E) Amber within matrix; orange and blue stripes are colors of amber under standard and UV light; scale bar 1 mm. (F) Amber within sedimentary matrix collected.

**Figure 2.**
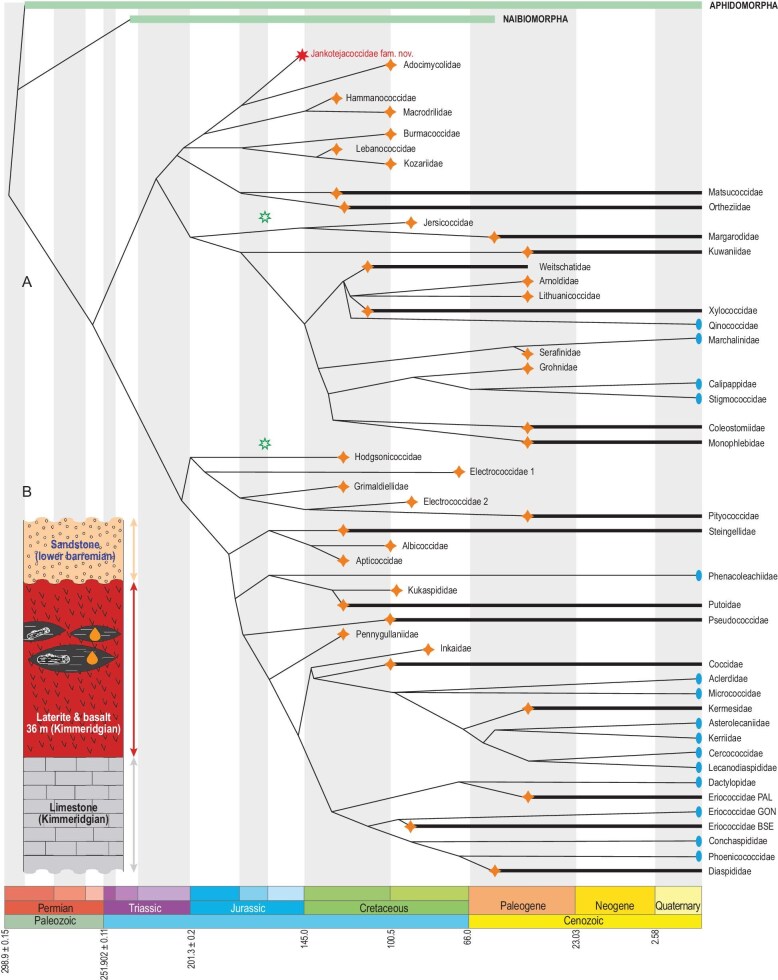
Dated phylogeny of Sternorrhyncha showing hypothesised relationship of the new family Jankotejacoccidae Szwedo, Azar et Sendi, fam. n.

**Figure 3.**
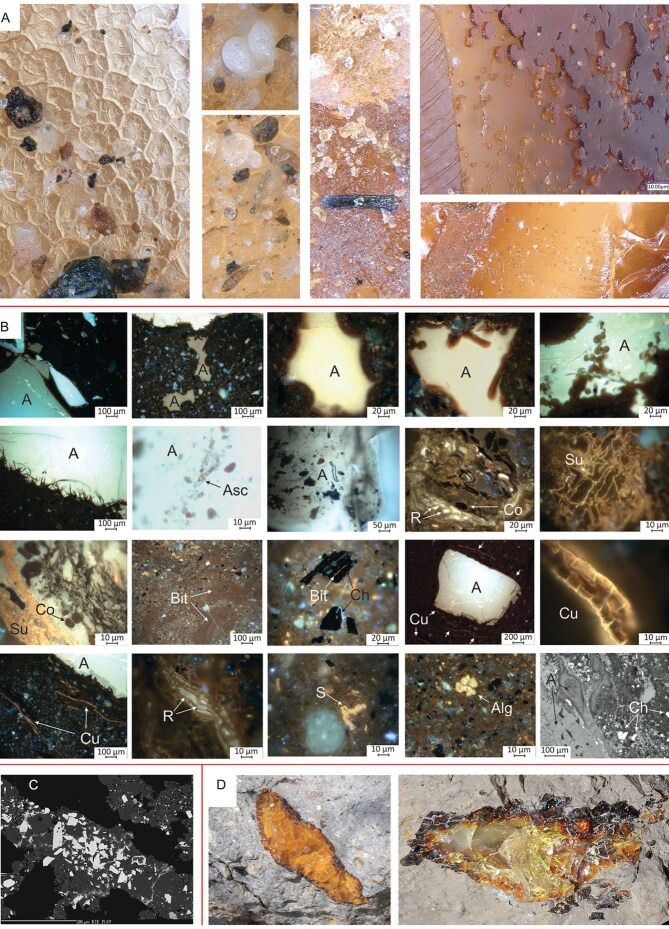
Details of sediment and boundaries between sediment-amber of Kimmeridgian Lebanese Aintourine amber. (A) Details on the amber revealing homogenous structures with rare bacterial traces and fungi. (B) Boundaries between sediment-amber and fossilized organic matter in sediment (explained in the text and Note S4: A—amber, Asc—fungal ascospore, Cu—cutinite, leaf cuticle, R—resinite, Alg—alginite, Ch—char, S—sporinite, Co—corpocolinite, Bit—bituminite, Su—suberinite) holotype SNM Z 40023B. (C) Back-scattered electron (BSE) image of laterite/bauxite sediment with böhmite groundmass (grey) and numerous grains of hydroxylian pseudorutile pseudomorphs after ilmenite (white). Goethite forms tiny irregular veinlets (pale grey). (D) Large amber particles.

**Figure 4.**
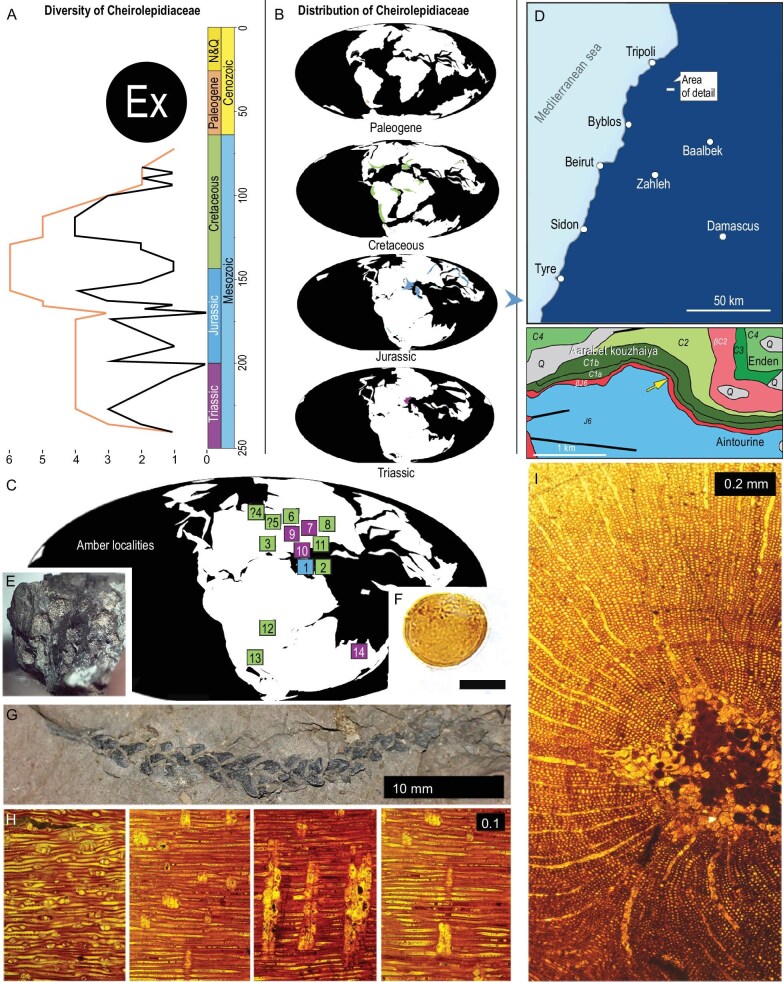
Localisation and revision of cheirolepidiacean forests over time. (A) Rangethrough (orange line) and sampled-in-bin (black line) diversity of Cheirolepidiaceae at genus level, including singletons. (B) Triassic—Paleogene paleogeographic maps with distribution of cheirolepidiacean forests over time. (C) Jurassic paleogeographic map with occurrences of Cheirolepidiaceae amber forests (1–2 Lebanon, 3 Spain, 4 UK, 5 France, 6 Germany, 7 Austria, 8 Slovakia, 9 Switzerland, 10–11 Italy, 12 Congo, 13 South Africa, 14 Australia). (D) Simplified map of Lebanon with localization of the studied area and its geology (modified after Dubertret and Wetzel 1951 and Wetzel and Dubertret 1945 [41,18]. J6 = Upper Jurassic; βJ6 = Basaltic Kimmeridgian (Upper Jurassic); C1a = lower Barremian (sandstone); C1b = Barremian; C2 = Jezzinian (uppermost Barremian—lowermost Aptian); βC2 = Basaltic uppermost Barremian—lowermost Aptian; C3 = Albian; C4 = Cenomanian; Q = Quaternary; faults; arrow for outcrop. Maps and data from https://paleobiodb.org using plate rotations ([42]; Tables S4–S5). (E) Cone. (F) Pollen of Cheirolepidiaceae *Classopollis* sp., same locality. (G) Leaf axis (22747–1) of amber-producing *Brachyphyllum* (same stratum, Khinchara, 33°55′8″N; 35°43′45″E; 960 m a.s.l.). (H, I) Radial and cross/tangential section (SW-33219–1.1–2) of amber-associated and amber-producing *Protopodocarpoxylon* wood (34°17′31.0″N; 35°56′39.8″E; 1290 m a.s.l.).

**Figure 5.**
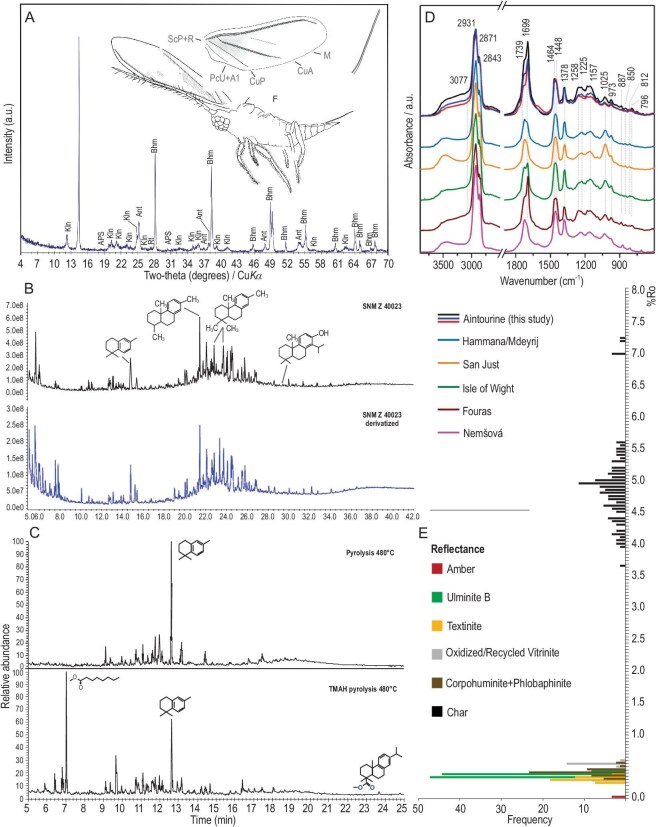
Sediment and amber analyses. (A) XRD analysis of the sediment (Ant = anatase; APS = aluminum-phosphate-sulfate mineral; Bhm = böhmite; Kln = kaolinite; Rt = rutile). (B) Total ion chromatograms of amber extract and derivatized amber extract. The list of identified compounds can be found in Table S1. (C) Total ion pyrograms of amber and derivatized amber (pyrolysed at 480°C for 20 s) showing the most abundant compounds. The list of identified compounds can be found in Table S2. (D) IR profiles for major cheirolepidiacean ambers (Aintourine, Lebanon; Hammana/Mdeyrij, Lebanon; San Just, Spain; Isle of Wight, UK; Fouras, France; Nemšová, Slovakia). (E) Reflectance histogram.

The science fiction film *Jurassic Park* influenced a whole generation of researchers, although having three major inconstancies related with ‘life in amber’ [1]: (1) DNA from the Mesozoic is decayed as well as blood [2], (2) there was no Jurassic amber known with insect inclusions [3], and (3) there was no evidence of biting insects in the Jurassic [4]. The latter two objections are no longer valid, and one of them entirely, as we describe here an insect inclusion in Late Jurassic (Kimmeridgian) amber from Aintourine in North Lebanon. Although instead of sucking blood, the fossil is of a plant sucker—an insect cohort known from the beginning of insect history [5]. The discovery (Figs 1–5) was enabled due to recent discoveries of 469 localities of amber within Lebanon [6], of which 19 are of Late Jurassic age [7]. Also documented are physical associations of amber with wood (e.g. *Brachioxylon* or *Protopodocarpoxylon*), pollen (e.g. Cyatheales and pollen related to the cheirolepidiaceans), and leafy axes (*Brachyphyllum* or *Frenelopsis*) (Fig. S6) of cheirolepidiaceans [8–12].

This first known fossil organism from Jurassic amber provides new insights into the early evolutionary history of Sternorrhyncha. Although amber has been known since the Carboniferous, organisms prior to the Cretaceous remain very scarce. This paper deals with one of the possible factors behind this. Furthermore with the help of a wide array of methods the precise age, amber source, and depositional environment of the fossiliferous Jurassic amber outcrop are revealed. New discoveries of organisms in Jurassic amber are promising and offer a window to past ecosystems from this time interval.

## SCALE INSECTS AND THE EARLIEST KNOWN FOSSIL IN AMBER

The earliest amber-preserved and herein described plant-sucking insect is of a hemipteran (Sternorrhyncha) (Fig. 2A). Hemipteran fossils date back to the Late Carboniferous [13]. Members of the sub-order Sternorrhyncha include modern aphids, whiteflies, jumping plantlice, and scale insects, as well as their extinct relatives. Extant scale insects are mostly less than 5 mm long and cryptic in habit, but include many important agricultural, horticultural, and forestry pests. Some secrete a waxy covering either as a structure detached from the body (a scale or test) or as a secretion that adheres to the body surface. They are the most species rich and morphologically diverse sternorrhynchans. Scale insects have a worldwide distribution, are classified in 57 families including 21 extinct [14,15] and are divided between the archaeococcoids (∼800 species) and the neococcoids (∼7500 species). Sternorrhynchan fossils have been known since the Early Cretaceous. Being phloem-feeders they damage plants directly through loss of sap, but the main damage they cause is indirect by facilitating microbial infections. Honeydew, the sugar‐rich waste from phloem-feeding, promotes the growth of sooty mold, which can stifle photosynthesis but is an important dietary component of insects, especially ants. Male scale insects display complete metamorphosis, whereas female development is paedomorphic (adults resemble nymphs). They display diversity in reproductive systems (e.g. parthenogenesis, hermaphroditism, and paternal genome elimination), chromosome number, sperm structure, and types of endosymbiosys [15–17].

Here the first ever amber fossil insect from the Jurassic, *Jankotejacoccus libanogloria* Szwedo, Azar et Sendi, gen. et sp. n. is described (Figs 1C, E, and 5A). It is a scale insect (Hemiptera: Sternorrhyncha), and thus a member of the fifth most speciose group of extant insects. We place it in its own family, Jankotejacoccidae Szwedo, Azar et Sendi, fam. n (Note S2). Finding this fossil shows that factors and processes resulting in the origin of characteristic features of the coccid male, influenced the morphological and behavioural shifts and modifications at very early stages of evolution, as the present Late Jurassic archaeococcid fossils of males appear to be rather ‘modern’ and could not relate to angiosperms absent at that time.

All scale insects are opophagous, feeding on phloem as other Sternorrhyncha, but feeding is limited to nymphs and females. Males with developed mouthparts do not feed and die within three days after emergence [18]. The ancestral forms of scale insects shifted to hypogeic habitats, resulting in diverging evolutionary traits in respective sexes, leading to dwarfism in males, their short appearance, and loss of functionality of mouthparts [19]. The new family is established based on rigorous comparative analysis of morphological features of this fossil and its potential relatives. All 150 characters available to infer from fossils and present in living taxa were checked, analyzed, and compared. Separation of features and their equivalence might seem obscure; many appear to have a random distribution, others display convergences. The present fossil reveals the states of 33 characters, and therefore support for character distribution analysis is low. We made the analysis of features of our Jurassic fossil with Early Cretaceous fossils from Lebanese amber, and with the addition of the recently described Macrodrilidae and Adocimycolidae from Burmese amber and living archaeococcids Ortheziidae and Qinococcidae [20]. The new family appeared in the clade with Adocimycolidae and Macrodrilidae, but support for this clade is very low and a number of homoplastic characters were revealed (Figs S7, S8; Notes S2, S8; Table S8; and Supplementary nexus file).

**Description of fossil (for details see Figs**  **1****C, E**, **5****A and**  Notes S2, S8)

Family *Jankotejacoccidae Szwedo*, Azar et Sendi, fam. n. urn:lsid:zoobank.org:act:549C19D9-1E2B-48C6-95AF-E548584BDA3C

*Jankotejacoccus Szwedo*, Azar et Sendi, gen. n. urn:lsid:zoobank.org:act:60EDC0A9-FFAA-48EF-89F6-3D4E37052389

*Jankotejacoccus libanogloria* Szwedo, Azar et Sendi, sp. n. urn:lsid:zoobank.org:act:B6A07A3F-0A31-4C33-AEC9-F6A0CA4D29FD (Fig. 1C, and E); Holotype, alate male, SNM Z 40023; deposited in the Slovak National Museum, Natural History Museum in Bratislava

Diagnosis: Alate male. Antenna 10-segmented (plesiomorphic condition, shared with Burmacoccidae Koteja, 2004, Kozariidae Vea et Grimaldi, 2015, and Alacrena Vea et Grimaldi, 2015), with scapus and pedicel not enlarged (pedicel conspicuous in Burmacoccidae, Kozariidae, and *Alacrena*), antennomeres III-X with long, protruding setae, arranged in whorls (similar pattern is observed in Hammanococcidae Koteja et Azar, 2008 and Weitschatidae Koteja, 2008; setae short in Burmacoccidae, fleshy and capitate in Kozariidae and *Alacrena*); compound eyes consisting of few ommatidia (similar to Burmacoccidae); fore wing hyaline, with wrinkled sculpture, with microtrichia only in anterior portion (in Burmacoccidae and Kozariidae fore wing with microtrichia; microtrichia absent in *Alacrena*), alar lobe present (as in Burmacoccidae); pterostigma weak, but present (pterostigma absent in Burmacoccidae, Kozariidae, and *Alacrena*); Sc + R shifted from margin, not reaching forewing tip (similar to Burmacoccidae); ‘rs’ [RP] and ‘afx’ [trace MP] absent; ‘cua’ distinct, long, reaching almost the margin (similarly long, but weakened in Burmacoccidae and Kozariidae); ‘pfx’ [posterior flexing patch, remnant of CuP] visible; clavus with vein Pcu(?) weak, but visible. Haltera present, elongate, narrow. Legs long, with rows of capitate setae, tarsal claw long and digitules present; abdomen tapering posteriad, long wax filaments from abdominal tergite 7 and 8, penial sheath short(?).

## GEOLOGICAL SETTING

The Late Jurassic amber of Aintourine (Zgharta District—North Lebanon), with the fossil insect inclusion, is found in a shale lens. The fossiliferous grey to black shale which forms a lens (Figs 1A, 2B, 4D) is rich in lignite surrounded by brown and reddish laterites that occupy pits in Kimmeridgian volcano-basaltic complex soil. During the Kimmeridgian (Late Jurassic), due to active paleotectonics, there was volcanic activity with basaltic effusions that created relief (sometimes even formed islands) in shallow marine water environments where the basaltic loams are associated with neritic sediments [21–23]. The water was agitated, while the volcanic products and within hollows in volcanic rocks, water was calmer, which allowed the vegetal and resin remains to accumulate. Sometimes the accumulation of lenses rich in lignite was caused by small faults that formed the relief. Lenses of laterites and lignite reached several dozens of meters in length with no more than 0.5 m thickness. The lenses are 2–3 m thick when they are in direct contact with faults or pits in basalt that allowed their accumulation, thus explaining the unusual association of basalt with lignite [23].

Lateral changes within the Kimmeridgian volcano-detritic complex of the Bhannes Formation reveal a combination of terrestrial and shore habitats. The volcano-basaltic outcrops (βJ_6_ in geological maps) were influenced by the seabed rising towards the surface and the appearance of small fractures. Magma followed them to reach the surface. An irregular topography due to volcanic inputs and fault activity, formed mainly in a shallow submarine environment, which included islands on the offshore of the tropical north-east of Gondwana. The material, composed of brown and red clay, is the result of degraded and altered lava that had been carried by the currents and has been widely distributed and associated with the deposits. Bhannes Formation has several lateral facies changes that vary (upon regions) from basalt columns (Bkaa Kafra, North Lebanon), basaltic pillow lava (Qahmez), to variable brown and red laterite (North Lebanon) and clay (Central and South Lebanon). Locally the clay contains lignite formed in isolated basins protected from basalt input and fault activity or on the shores of small islands. Jurassic lignite is always arranged inside short clay lenses that locally become thick towards the basalt barrier or fault mirror on which they are accumulated, or in stream channels on islands. The localities with lignite that contain amber [7,23] are mainly in North Lebanon. *Brachyphyllum* leaves are present in Daher El-Sawan (2 localities); Qornet Chahwan; Beit Chabab and Khinchara in Central Lebanon, associated with Jurassic marine shells, shrimps, *Asteracanthus magnus* teeth (in Wata el Jaouz), and specifically common stratigraphically important Kimmeridgian spines of *Pseudocidaris mammosa* sea urchin [24] and undiagnostic (D-)epibysate bivalves *Modiolus/Inoceramus*.

## ORGANIC PETROGRAPHY, REFLECTANCE ANALYSIS, XRD, AND EPMA

Isomorphic admixtures of primary ilmenite in the Lebanese sample, accommodated into a pseudorutile structure, represent laterite or bauxite, Al-rich residuum probably after a magmatic extrusion was subjected to intense chemical weathering under tropical or subtropical conditions [e.g. 25]. In the laterite there might be a relief, which favored the deposition of leaves, wood, and amber in a dead current zone. Lack of quartz may indicate either redeposition or a quartz-free parental rock. Hydroxylian pseudorutile is a product of low-temperature, supergene weathering of primary ilmenite connected with its oxidation, hydration, and partial leaching of iron [26,27] probably during formation of laterite/bauxite. Common presence of Mg > Mn, V-bearing pseudorutile after primary ilmenite, corroborates a rather basic magmatic rock precursor of basaltic composition.

The depositional environment of the amber-bearing sediment was studied under reflected-light microscopy (Fig. 3, Note S6). Boundaries between amber and sedimentary rock have the shape of straight lines (Note S4) or curved contours (Note S4 and Fig. S1). Narrow and sharp edges suggest that brittle (broken) resin got into the sediment, while semi-angular edges suggest penetration of low-viscosity resin into empty or partly waterlogged cavities. Low viscosity can partially explain the scarcity of insect inclusions within Jurassic amber, probably combined with a paucity of large resin producers. In some cases, there are rod-shaped or disc-shaped structures on the edge of the individual pieces of amber that have a reddish-brown color under UV light (Note S4, Figs S1.4, 5, and 6). These shapes probably formed when the low-viscosity resin fell into the aqueous environment. Frequently the amber is wrapped in a few layers of orange-fluorescing cutinite (fossil cuticles derived from leaves), suggesting that the resin fell on the surface covered by *Brachyphyllum* leafy axes (Note S4, Fig. S2). The amber is usually clear, and inclusions of fungal ascospores (Fig. S1.7), mineral and organic debris, e.g. char, leaves, woods, and fungal tissues (Note S4 and Fig. S1.8) are rare in the marginal parts of the amber (Figs 1F, 3A, 3D). There are no indications of longer transport and re-sedimentation of the amber, so the depositional environment was most likely located close to the resin-producing tree. The amber-bearing sediment contains organic residues mainly originating from higher plants (Note S4, Fig. S2.9–17, and 20), as well as fossilized sapropel and a very small portion of algae, indicating an occasionally waterlogged environment near the forest, forested swamp peatland, or peatland edge forest. Litter from vegetation was deposited together with acidic fine-grained lateritic soil, developed by intensive and prolonged weathering of the underlying Kimmeridgian basalts. Over time, a peat swamp developed at this site and a part of the organic debris mixed with the soil which then underwent microbial decomposition in a low oxygen aqueous environment. This is supported by a higher amount of fossilized sapropel (bituminite-III), which was formed under reducing conditions by microbial degradation of organic debris. Increased amounts of specific parts of vascular plants, such as bark of branches and roots, leaf cuticles and resins (macerals suberinite, cutinite, and resinite) may be the result of concentration due to selective preservation, as these are more resistant to microbial degradation. Pollen grains as sporinites (Fig. 1D, Fig. S2.18) and *Botryococcus* algae (Note S4 and Fig. S2.19) are also present in the sediment. Absence of pyrite suggests that the site was isolated from sea water during deposition [28]. Based on mean reflectance (Fig. S3) of maceral ulminite B (0.4%Ro, Fig. 5E) the maximum burial temperature of amber and amber-bearing sediment probably did not reach 40°C throughout their whole geological history [29]. Heat-modified char particles found in the sediment (Note S4, Figs S2.13 and S20) suggest that a ground paleo-peat fire occurred at some distance from the amber deposition site. Based on various studies [30–35] and the average reflectance of char particles (4.68%Ro, Fig. 5E), temperatures during a paleo-peat fire could have reached 595–800°C (Note S5, Table S3).

## ANALYSIS OF AINTOURINE AMBER—(PYROLYSIS) GC/MS ANALYSIS

Jurassic Aintourine amber revealed cheirolepidiacean chemistry. The Aintourine amber is mostly transparent, homogenous, pale orange-pale yellow to mainly red, and it seems entirely empty (Note S3, Figs 1B, F, 3A–B, D, 5B–E, Tables S1 and S2). In the extractable organic fraction, non-phenolic abietane derivatives were the major components, including the most abundant 16,17,18-trisnorabieta-8,11,13-triene. Lower amounts of n-alkanes, pristane, totarol, and pimarane derivatives were also detected. The non-phenolic abietanes are the largest class of tricyclic diterpenoids widely distributed among conifer families [36]. The identified derivatives are not those found in fresh resin, and are the results of aromatization and oxidation processes that proceeded in sediments under aerobic conditions (due to lack of phytane and presence of aerobic-specific pristan). Totarol, a member of the phenolic abietane group, is considered as a chemotaxonomic marker for Cupressaceae and Podocarpaceae. Moreover, analysis of a derivatized extract revealed esters of fatty acids and other oxygen-containing compounds, e.g. 16,17-dinorcallitrisic acid methyl ester. The occurrence of this compound is limited to Cupressaceae [37]. Pyrolysis showed that the dominant compound is ionene, together with abietanes and alkyltetralines, and fatty acid esters. Ionene is a diagenetic product from labdanes, evidencing intensive degradation processes. The possible source seems Cupressaceae or Podocarpaceae. Diagenetic processes obscure the identification, and there doesn't need to be a modern analogue to determine the specific biomarkers to the possible cheirolepidiacean representative. The analytical record is similar (in the general pattern of pyrolysis as well as identical 16,17,18-trisnorabieta-8,11,13-triene, totarol, pimaradiene, and very similar compounds [38]; bicyclic products and abietan types and analogical, derived, and isomere compounds [39]) to records for the Early Cretaceous ambers from Spain (associated with cheirolepidiacean *Frenelopsis*) [38], Fouras in France (associated with cheirolepidiacean *Pagiophyllum* Heer, emend. Harris, 1979) [12], and the Isle of Wight in southern England (dominated with cheirolepidiaceans) [39], suggesting Cheirolepidiaceae as one of the possible botanical sources. This is an extinct family (259.0 to 61.7 Ma) similar to Cupressaceae. These analyses were supported by macrofossil evidence associated directly with the amber, by *Protopodocarpoxylon* wood, *Brachyphyllum* leaves, *Classostrobus* cones, and *Classopolis* pollen, like in Cenoman-Turonian amber deposits from France [e.g. 9,12].

## IR SPECTRA

IR spectra of studied Jurassic ambers are unique (Fig. 5D, Figs S4, S5, and Table S7), and differ significantly from spectra for Cretaceous Lebanese ambers, e.g. Hammana-Mderyij. This is likely due to their shorter deposition as their origins are expected to be of the same cheirolepidiacean trees (see above and below). Variations are clearly visible in the spectral region attributed to the stretching mode of hydroxyl groups near 3400 cm^−1^, displaying higher intensity of the OH absorption bands for the herein studied amber compared to amber from Hammana-Mderyij. A distinction can be also observed in the more pronounced intensity of carbonyl groups with characteristic bands (1790–1650 cm^−1^) relative to the complex bands of stretching and bending C–H vibrations of aliphatic hydrocarbons (maxima near 2930, 1464, or 1378 cm^−1^). This feature is reflected by the higher content of esters or carboxylic acids species in the studied Jurassic amber compared to amber from Hammana-Mderyij and other selected ambers from San Just (Spain), the Isle of Wight (UK), Fouras (France), and Nemšová (Slovakia) (Fig. 5D). In general, the IR profiles of Cretaceous Lebanese amber are highly congruent with IR profiles of European ambers. Their similarities indicate the same origin from cheirolepidiacean trees (see paragraph ‘Comparison of ambers’). A similar IR pattern is also observed in the Cedar Lake amber (Canada) of uncertain origin [40]. However, spectra of Fouras amber (France), have a different shape, especially in the spectral region in which characteristic bands of carbonyl groups occur, i.e. ∼1700 cm^−1^, despite its confirmed origin from cheirolepidiacean trees. Regarding the IR spectroscopy of only Jurassic ambers, there is wide variability between closely associated sites, caused by the different age and post-depositional modifications, with similar spectral patterns for Aintourine samples [7]. The shape of bands differed in the 1300–950 cm^−1^ spectral region. Variations from spectra of amber studied here could be attributable to the presence of accessory minerals that might have influenced the shape of spectra, especially in the finger print region below 1300 cm^−1^ due to overlapping with bands present in the ambers. However, those bands are not observed in such intensity here. Among reported Jurassic ambers, amber of Harissa in Lebanon revealed an almost identical spectral profile, considering the presence and positions of absorption bands (Fig. S4A–C).

## CHEIROLEPIDIACEAE

Cheirolepidiaceae are a family of conifers consisting of 15 genera of which 9 are known from large fossils, documented by 924 fossil records [43,44]. Its first unequivocal evidence is *Frenelopsis* Schenk, 1869 in the Ladinian sediments of South Tyrol, Italy, while last occurrences are documented by Paleocene *Classopolis* Pflug, 1953 palynomorphs in San Ramon, Argentina and Subeng, China [40]. They were thermophilous, geographically widespread and of great paleoecological importance, especially in the Jurassic and Early Cretaceous at low paleolatitudes (Fig. 4A–C). Their radiation is associated with the dominance of frenelopsid forms [45], particularly in southern Laurasia and northern Gondwana (Lebanon at that time was in NE Gondwana). The fossil record of Aintourine includes primarily cuticular remains, and also seed cones and pollen. Amber from the trees remains scarce (Fig. 4C). After the K1/K2, their diversity declined significantly [44] and they become replaced by angiosperms near the K/Pg boundary [46] as well as by other contemporary conifers. Entomophilous pollination has been suggested for some of the representatives [47]. Cheirolepidiaceae adapted to a wide range of environments, including maritime, forming extensive coastal forests near (sub)tropical seas, but also freshwater lakes and rivers [46]. Some representatives tolerated poor sandy soils, while others adapted to better drained soils [46]. Their morphological and anatomical diversity suggest high degrees of biological specialization [48]. High percentages and large morphological variety of pollen in the respective pollen spectra as well as recurrent associations with other types of miospores [49–51] support a variety of ecological niches [45,52]. Evidence for their radiation during the Jurassic is mainly based on the numerous species of vegetative shoots (e.g. *Frenelopsis, Pseudofrenelopsis*) and a significant quantitative increase in pollen in many Gondwanan locations [53,54]. The virtual absence of medium-sized insect fossils in cheirolepidiacean ambers from Nemšová, the Isle of Wight, Spain as well as Cretaceous Lebanese ambers may reflect the absence of large resin production, unsuitable resin chemistry for preservation of biological inclusions, and the low viscosity of cheirolepidiacean amber.

## PLANT MATERIAL PRESERVED ALONG THE AMBER

Wood of *Protopodocarpoxylon* Eckhold, 1922 [e.g. 45] preserved along with Aintourine amber is characteristic for Cretaceous Lebanese ambers (Fig. 4H and I). It is homoxylous with indistinct growth-rings and possesses rare axial parenchyma. A pith is present, indicating a small branch or narrow stem. There is a 1–2-seriate mixed pitting of the radial tracheid walls, and the pits are often contiguous. The rays are very low, generally up to 4-cells high. Finally, there are 1–3 podocarpoid cross-field pits (with half-bordered to almost reduced borders) per field. Both horizontal and end (tangential) walls of the ray parenchyma are thin and smooth. Ray tracheids and resin canals are absent. All are diagnostic features for Cheirolepidiaceae [55] and chemical analysis excludes an araucarian source. Indeed, it has recently been suggested that ‘araucarian’ *Agathoxylon* wood could actually represent a cheirolepidiaceaen [56,57], and this might explain why Myanmar amber is claimed to be araucarian or cupressacean in spite of having a chemically different origin [58], which is supported here as well. *Brachyphyllum* leafy axes originating from the amber-producing tree were also preserved, locally in high numbers, in association with the amber (including holotype), and carbonised and decomposed to small particles. Whole cheirolepidiacean leafy axes were preserved in adjacent localities of the same strata (Fig. 4G). Cone fragments were also recorded in the holotype (Fig. 4E).

## PALYNOLOGY

Thirteen different palynological types (Note S7, Table S6) were recovered from the amber-bearing strata. The taxonomic richness is lower than generally found in Cretaceous sediments [50]. The poor preservation of the palynological material suggests a complex pre-burial history, probably involving site-specific, obscure taphonomic processes (lateritization is connected with chemical weathering of underlying rocks where the soil first starts to be acidic with a high oxidizing capacity and later are alcaline with reducing capacity). Although some long-distance airborne input is likely, modelling and pollen trap studies in modern (sub)tropical forests indicate that pollen from such areas tend to be nearly exclusively from surrounding vegetation [59–61]. Thus, the dominance of spores (38% of the 91 identified specimens of miospores, see Note S7) including extant tree fern families (Cyatheales) and pollen related to the cheirolepidiaceans (38%, see Note S7, Fig. 4F) offer an accurate picture of the local vegetation, which complement and support the data provided by mega- and mesofossils. Considering the presence of bauxite and laterite, this co-dominance of the two types of miospores reflects forested vegetation including a large proportion of the aforementioned elements growing under a high, but probably strong seasonal, precipitation pattern [44,62]. Cheirolepidiaceans and tree ferns are represented in younger amber-bearing settings, and also characterized by seasonal variation of moisture.

## CHEIROLEPIDIACEAN AMBER SOURCE AND ITS IMPLICATIONS

The cheirolepidiacean amber source described here enables a better understanding of the origin and evolution of cheirolepidiacean amber-producing trees. Wood preserved along with amber and chemical analysis reveal a *Protopodocarpoxylon* source, here unequivocally identified as Cheirolepidiaceae. Amber probably from the same source and locality, but of significantly younger age (Barremian, Early Cretaceous), is superficially similar, but due to longer deposition of Aintourine amber, more organic compounds were changed and thus results of IR spectroscopy and pyrolysis differ (see above; Fig. 5D, Figs S4–S5). By analogy and IR profiles, Cretaceous Lebanese ambers can be directly linked with several ambers from Slovakia, France, and Spain (see Material section for details on localities) of cheirolepidiacean origin (Note S7). Chemical and structural changes meant that in some cases, as with the amber from the Isle of Wight in UK, with IR and GC MS pyrolysis indicated an obscure origin (with pineacean evidence based on absence of decayed characteristic cheirolepidiacean chemical compounds), although they are also of cheirolepidiacean origin (supported with preserved macrofossils [39]). The amber-producing trees were not necessarily representative of the dominant forest trees. By analogy, copal-producing trees in Ecuador constitute less than 4% of the trees in the forest, but copal is found all along this forest down to 4 m depth, even on surfaces where these trees do not currently grow. Apparently it is sufficient for copal deposition if copal trees lived in the area once a thousand(s) years ago (UNESCO BR Sumaco, Ecuador, 2015; 0°25′59.8″S, 77°19′58.4″W, 972 m a.s.l.). Cheirolepidiacean trees are frequently (also in this case) associated with fossils of araucarians and these two groups likely coexisted during most of their Mesozoic history.

## CONCLUSION

The first known fossil organism from Jurassic amber, *Jankotejacoccus libanogloria* gen. et sp. n., is herein described, a plant-sucking adult male scale insect, differing greatly from all other coccids, and therefore placed in a new family. The fossil reveals that characteristic features of the coccid male, including morphological and behavioural shifts and modifications occurred at a very early stage of coccid evolution, since the Late Jurassic archeococcid fossils of males seem to be morphologically related to extant taxa and are associated with gymnosperms, which were dominant at that time. A Kimmeridgian age was supported by strata-associated marine organisms, and was also revealed by zircons. Aintourine amber is shown to be of cheirolepidiacean origin. The low-viscosity resin, suggested by semi-angular edges of boundaries between sediment-amber, might be one of the factors that led to only rare preservation of organisms in this type of amber, although other factors, such as flow resin production or other factors affecting the fossilization process, might have also played a role. Associated plant material of Aintourine amber included the cheirolepidiaceans *Protopodocarpoxylon, Brachyphyllum, Classostrobus*, and *Classopoli*s pollen, and the paleoenvironment was reconstructed as a forested temporary swamp habitat with tall araucarian, ginkgoacean trees and shrub ferns, tree ferns, and water ferns. The depositional environment consists of (sub)tropical lateritic soil with vegetation debris that underwent incomplete microbial decomposition in an anoxic water environment of peat swamp development (due to presence of algae, higher plants, and sapropel). Lack of pyrite indicates that during deposition the site was isolated from the sea. The presence of bauxite and laterite reflects forested vegetation with a probably strong seasonal precipitation pattern. Char particles discovered in the surrounding sediment of the amber were modified by heat and suggests that paleo-peat fires were present at a distance from the deposition site. The amber is usually transparent with several inclusions of fungal spores, mineral and organic debris (char, leaves, woods, and fungal tissues). There are no indications of long-distance transport and re-sedimentation of the amber, so the depositional environment was likely located in the vicinity of the amber-producing trees. Nevertheless, it remains questionable if the amber-producing trees were dominant in these forests, and they probably coexisted with other trees. Fossil organisms in amber are usually exceptionally well preserved, and new discoveries from the Jurassic are crucial to gain knowledge on the evolutionary history of terrestrial lineages, and a better understanding of dynamic past ecosystems.

## MATERIALS AND METHODS

### Locality

The fossiliferous Kimmeridgian amber outcrop (discovered by D.A. and Raymond Gèze in 2009) is situated in the small village of Aintourine (Figs 1A, and 4D), Mouhafazet Loubnan Esh-Shemali (= Governorate of North Lebanon), Caza (= District) Zgharta; at the beginning of Aintourine village on the road leading from Ehden to Aintourine (34°17′31.0″ N; 35°56′39.8″ E; 1290 m a.s.l.). The outcrop is situated in a locality named Ez-Zouarib on the left side of a small road, before arriving to the first houses of Aintourine, and beneath the western side of the town of Ehden. The amber fragments, translucent red to orange and transparent yellow in color, are of various sizes and include some exceptionally large pieces (∼3–5 cm in diameter) in lignite, dark shale, and clay mixed with laterites, and located on a Kimmeridgian basaltic deposit [7].

### X-ray powder-diffraction (XRD)

The diffraction data was analysed using DIFFRAC.EVA version 4.2.1 software [63] coupled to the PDF-2/2010 version of the International Centre for Diffraction Data [59] database (see Note S1: Materials and Methods for details).

### Gas chromatography/mass spectrometry (GC/MS) analyses

Data processing was carried out using Chromeleon software (Thermo Scientific) (see Note S1: Materials and Methods for details).

### Pyrolysis-gas chromatography-mass spectrometry of amber

For data processing, Xcalibur software (ThermoElectron) was used. Components were assigned from retention times and comparison with mass spectra from the National Institute of Standards and Technology spectral library and literature [64]. The content of an individual compound was expressed as the relative abundance in percentage of the total area: the area of the individual peak was divided by the total area of the integrated total ion current.

### Infrared spectra of amber

Spectra were measured and operated using OMNIC^TM^ software.

### Organic petrographic and reflectance analyses

Organic petrographic and reflectance analyses of amber and adjacent sedimentary rock were performed. Organic petrographic examination on a polished thin section under incident white and UV light was carried out to determine a maceral composition and the amber characteristics using a Zeiss AX10 microscope system. Maceral descriptions and terminologies for vitrinite, huminite, and liptinite follow [65,66], respectively, and for char particles [67,68] (see Note S1: Materials and Methods for details).

### Electron-probe microanalysis (EPMA)

Detection limits of the measured elements were 0.02 to 0.08% wt. The matrix effects were corrected using the PAP procedure [69] (see Note S1: Materials and Methods for details).

### Palynological analysis

Palynological material (up to 25 g) results from the maceration of sedimentary material in HF, HNO_3_, and HCl, followed by filtration with 8 micron mesh.

### Microscopy

Optical investigations were performed using LEICA DMV6 equipment.

### Parsimony

The nexus file was prepared with Mesquite 3.91 build 955 [70]. Trees were calculated with TNT 1.6 [71] and analysed with ASADO 1.85 [72], traditional search and new technology searches were performed with both equal and implied weighting [73,74], with different values of the k parameter [75]. The most parsimonious trees with the same topology, were received in both analyses, with Implied Weighting with k parameter 12 for fossils and missing data treatments. Unambiguous Changes Only, Slow Optimization and Fast Optimization [72,76] trees were analyzed. The most parsimonious tree is 103 steps long Ci = 52 Ri = 63.

### Deposition

Samples SNM Z 40023ABC (holotype; a slice for sediment-amber analysis; and a slice for mineralogical analysis) are deposited in the Slovak National Museum, Natural History Museum in Bratislava and was (SNM Z 40023A, after analyses) embedded in epoxy resin and cut to a small piece 18 × 6 ×2 mm (see Note S1: Materials and Methods for details).

## Supplementary Material

nwae200_Supplemental_File

## DATA AND MATERIALS AVAILABILITY

All data used in this study is available within the published version of the manuscript and in supplementary materials. The Life Science Identifier (LSID) for the new genus and species has been deposited at ZooBank: Publication LSID: urn:lsid:zoobank.org:pub:87DF9943-302E-47F3-9A96-D69C14D860A1.
