# Supplementary material for: Jurassic Park approached: a coccid from Kimmeridgian cheirolepidiacean Aintourine Lebanese amber
Source: Natl Sci Rev. 2024 Jun 11;12(3):nwae200. doi: 10.1093/nsr/nwae200 (PMC11895504; doi:10.1093/nsr/nwae200)
Supplement: nwae200_Supplemental_File [file nwae200_supplemental_file.docx]

Supplementary Data

**Jurassic Park approached: a coccid from Kimmeridgian cheirolepidiacean Aintouarine Lebanese amber**

Peter Vršanský, Hemen Sendi, Júlia Kotulová, Jacek Szwedo, Martina Havelcová, Helena Palková, Lucia Vršanská, Jakub Sakala, Ľubica Puškelová, Marián Golej, Adrian Biroň, Daniel Peyrot, Donald Quicke, Didier Néraudeau, Pavel Uher, Sibelle Maksoud, Dany Azar

Corresponding authors: [geolvrsa@savba.sk](mailto:geolvrsa@savba.sk); danyazar@ul.edu.lb

**The PDF file includes:**

Supplementary Text

Supplementary Figures 1 to 8

Supplementary Tables 1 to 8

Supplementary References

**Other Supplementary Data for the manuscript include:**

Supplementary Data 1.nex

**Supplementary Text**

**Supplementary Note 1:** Materials and Methods

X-ray powder-diffraction (XRD)

X-ray powder-diffraction (XRD) analysis of the amber bearing sediment was performed using a Philips PW 1710 diffractometer (Earth Science Institute SAS, Bratislava) under following conditions: Cu-anode (CuKα); secondary graphite monochromator, and proportional Xe detector, 40kV voltages; 20mA current; measurement extent of 4−70° 2Θ (step 0.02° 2Θ, 0.8 s/ step), divergent slit 1°, receiving slit 0.2 mm, primary and secondary Soller slits.

Gas chromatography/mass spectrometry (GC/MS) analyses

Gas chromatography/mass spectrometry (GC/MS) analyses of powdered samples (3 mg) dissolved in dichloromethane were performed using a Trace 1310 GC equipped with an ISQ single quadrupole MS (Thermo Scientific) instrument equipped with a TR-5MS column (60 m × 0.25mm × 0.25 μm). The oven temperature was from 40 °C (1 min) to 120 °C (15 °C/min), to 220 °C (6 °C/min), to 300 °C (5 min) (12 °C/min). The samples were injected in splitless mode, with an injector temperature of 250 °C and helium was the carrier gas. Mass spectra were obtained by scanning from m/z 45 to 650 in full scan mode. Aliquotes from the total extraction were converted to ester derivatives by a reaction with methanol and 14% boron trifluoride (BF_3_) for 1 h at 90°C and analysed using the same conditions.

Organic petrographic and reflectance analyses

Reflectance measurements were conducted using a LEITZ MPV-2 compact microscope photometer equipped with 50× oil immersion objective lens. Results are presented as mean random reflectance values (%Ro). From the appropriate population of macerals, the average value and standard deviation were calculated. Reflectance of huminite–ulminite B was used as a parameter of rank.

Pyrolysis-gas chromatography-mass spectrometry of amber

Pyrolysis-gas chromatography-mass spectrometry of amber (Py-GC/MS) was performed at 480 °C for 20 s, using a CDS Pyroprobe 5150 (CDS Analytical, LLC, USA) pyrolysis unit connected to a Trace GC Ultra-DSQ II (ThermoElectron) instrument equipped with a TR-5MS column (60 m × 0.25mm × 0.25 μm). The analyses were carried out with a temperature program from 40 °C to 300 °C at a heating rate of 8 °C/min. Pyrolysis was combined with methylation using a 25% (w/w) solution of tetramethyl ammonium hydroxide (TMAH-Py-GC/MS) (Sigma-Aldrich).

Infrared spectra of amber

The infrared spectra of amber were obtained on a Nicolet 6700 Fourier Transform Infrared (FTIR) spectrometer (Thermo Scientific^TM^). The set-up of the spectrometer was: IR source, KBr beamsplitter and DTGS detector were used for measurements in the middle IR region (MIR 4000–400 cm^-1^), transmission spectra were collected using a KBr pellet press technique (a small amount of the sample was homogenized with 200 mg KBr). The spectrometer and the chamber were purged with dry air. For each sample and measurement 64 scans were recorded with a resolution of 4 cm^-1^.

Electron-probe microanalysis (EPMA)

Electron-probe microanalysis (EPMA) was employed to analyse the mineral composition of laterite/bauxite using a CAMECA SX-100 apparatus (Dionýz Štúr State Geological Institute, Bratislava, Slovakia) under wavelength mode, 15 keV acceleration voltage, 20 nA sample current, 3 μm beam diameter, using natural and synthetic silicate and oxide standards.

Deposition

Wood and palynological slides SW-33219-1.1-2; SP-33219-1.1-4 and leaf axis 22747-1 (Khinchara, Matn District, Mount Lebanon Governorate, Central Lebanon) are deposited in the Lebanese University of Beirut, Lebanon. Investigation using syrup submersion was not possible due to the brittle character of the amber sample**;** similarly ST (epoxy embedding) was also impossible.

Cretaceous cheirolepidiacean ambers used for comparison (selection among ca. 200 newly analyzed worldwide ambers) originated from Barremian amber of Hammana/ Mdeyrij, Caza Baada, Mouhafazit Jabal Libnen [1], Lebanon; Barremian of Isle of Wight, UK [2]; Albian of San Just in Spain [3], Cenomanian of Fouras, France [4] and an undescribed Albian locality from Slovakia (all belonging to the Hammana/ Mdeyrij IR profile type although French Fouras among cheirolepidiacean ambers is even more similar to North American amber of North Carolina).

**Supplementary Note 2:** **Systematic description and discussion on the systematic position of the first Jurassic amber insect**

**Order Hemiptera Linnaeus, 1758** [5]

**Suborder Sternorrhyncha Amyot et Audinet-Serville, 1843** [6]

**Infraorder Coccinea Fallén, 1814** [7]  **[Coccidomorpha Heslop-Harrison, 1952]** [8]

**Superfamily Orthezioidea Amyot et Audinet-Serville, 1843** [6] **[Paleococcoidea: Borchsenius 1950** [9] **; Archaeococcidea: Bodenheimer 1952** [10]**; Orthezioidea *sensu* Koteja, 1974]** [11]

**Family Jankotejacoccidae Szwedo, Azar et Sendi, fam. n.** urn:lsid:zoobank.org:act:549C19D9-1E2B-48C6-95AF-E548584BDA3C

**Type genus: *Jankotejacoccus* Szwedo, Azar et Sendi, gen. n.**

**Diagnosis: Alate male. Antenna 10-segmented (plesiomorphic condition, shared with Adocimycolidae Poinar et Vega, 2023, Burmacoccidae Koteja, 2004, Kozariidae Vea et Grimaldi, 2015 and *Alacrena* Vea et Grimaldi, 2015), with scapus and pedicel not enlarged (as in Adocimycolidae pedicel conspicuous in Burmacoccidae, Kozariide and *Alacrena*), antennomeres III-X with long, protruding setae, arranged in whorls (long setae in whorls present also in Adocimycolidae; similar pattern is observed in Hammanococcidae Koteja et Azar, 2008 and Weitschatidae Koteja, 2008; setae short in Burmacoccidae, fleshy and capitate in Kozariidae and *Alacrena*); compound eyes consisting of few ommatidia (similar to Burmacoccidae); forewing hyaline, with wrinkled sculpture, with microtrichia only in anterior portion (in Burmacoccidae and Kozariidae forewing with microtrichia; microtrichia absent in Adocimycolidae and *Alacrena*), alar lobe present (as in Burmacoccidae); pterostigma weak, but present (weak pterostigma present in Adocimycolidae; pterostigma absent in Burmacoccidae, Kozariidae and *Alacrena*); Sc+R shifted from margin, not reaching forewing tip (Sc+R distinctly shifted from margin in Adocimyclidae; similar pattern as in Burmacoccidae); ‘rs’ [RP] and ‘afx’ [trace MP] absent; ‘cua’ distinct, long, reaching almost the margin (‘cua’ weak, not reaching margin in Adocimyclidae; similarly long, but weakened in Burmacoccidae and Kozariidae); ‘pfx’ [posterior flexing patch, remnant of CuP] visible; clavus with vein Pcu (?) weak, but visible. Halterae present, elongate, narrow.**

**Legs long, with rows of capitate setae, tarsal claw long and digitules present;**

**Abdomen tapering posteriad, long wax filaments from abdominal tergite 7 and 8, penial sheath short (?).**

**Description: as for genus.**

**Systematic remarks:** Scale insects (Coccinea), with over 8,400 species, are highly diverse, and most of them are small, cryptic, obligatory plant parasites often of economic importance. The diet of most of them is phloem sap. In addition to damaging plants by sap consumption, scale insects damage plants indirectly by secreting honeydew, which encourages the growth of sooty molds and interferes with photosynthesis. Scale insects also vector plant diseases [12]. Many unusual features of their morphology, reproduction strategies with diverse reproductive genetics including thelytoky (unfertilized eggs grow up female), arrhenotoky (unfertilized eggs grow up male), paternal genome elimination (descendant males do not express paternal genes), hermaphroditism, and life histories, with variable host specificities (specialists and generalists), and intimate relationships with endosymbionts and social Hymenoptera make them to be considered as some of the most evolutionarily fascinating organisms amongst insects [13–16].

**The new fossil described above presents a very ‘modern’ set of features, similar to those observable among living male representatives of scale insects. The decreased number of ocelli forming compound eyes is a derived condition, but present also in the Early Cretaceous Lebanococcidae, mid-Cretaceous Burmacoccidae, Kozariidae, Late Cretaceous Jersicoccidae, while the Early Cretaceous and late diverging Ortheziidae have normal multifacetted compound eyes. The antenna consisting of 10 antennomeres is a plesiomorphic condition in all Sternorrhyncha, and it is retained in numerous fossil (Burmacoccidae, Kozariidae, Jersicoccidae) and living archoeococcids. An interesting feature of *Jankotejacoccus* gen. n. is the presence of long setae on the antennae, distinctly longer than those in Burmacoccidae, resembling those of Jersicoccidae. Thoracic structures in *Jankotejacoccus* gen. n. are not well preserved, but resemble the general model of archeococcids. The structure of the forewing of Jankotejacoccidae fam. n. is similar in Burmacoccidae, with the alar lobe present, Sc+R shifted from margin, not reaching the forewing tip as in Burmacoccidae, ‘cua’ distinct, long, reaching almost the margin, but stronger marked than in Burmacoccidae and Kozariidae. The presence of weak pterostigma also seems to be rather a plesiomorphic condition, as the pterostigma is present in Aphidomorpha and Naibiomorpha, but among archeococcids it is present e.g., in Jersicoccidae and Margarodidae. The legs are relatively long, as in the majority of archoecoccids, covered with setae, with capitate digituli and with a single claw – such structure of leg is very late-diverging, resembling the patterns observed in males of living scale insects. The abdomen and genital structures are weakly visible, but in general resemble those observed in the Cretaceous representatives of archeococcids.**

**The new fossil described above is the first pre-Cretaceous record of scale insects. The Cretaceous fossil record reveals that the group differentiated early on and was already diverse, but the earlier stages of the scale insect evolution are virtually unknown.** The oldest two males of Coccinea, found among Upper Jurassic insects of Kazakhstan, bear a few simple eyes and probably one is a neococcid, while the other could be associated with ortheziids [17–18]. **As suggested by Koteja** [19] **and supported by the total-evidence approach of Vea and Grimaldi** [18] **origins of scale insects should be pushed back to the Triassic.** Koteja [19] suggested that ancestral coccids could shift to “hypogeic” habitats, on the litter of the forest floor. Such behaviour and habitat is still present in archaeococcids, e.g. Ortheziidae, and Margarodidae [20–22] as well as in some neococcids, e.g. Micrococcidae, Pseudococcidae, Rhizoecidae [23–27]. The Late Triassic–Early Jurassic Coccinea (alas unknown) were very probably associated with gymnosperms and these ancestral forms died out [19,28–32]. The Late Triassic is well documented as the ‘heyday of gymnosperms’ [33–35]. However, their highest net diversification may have taken place slightly earlier, in the Middle Triassic, followed by significant changes of origination rates across the Triassic–Jurassic boundary [36]. Recent work suggests that insects also started to show a shift towards modern forms sometime during the Triassic [37]*,* it was the time of the initial diversification of many groups, e.g. hemipterans, beetles, dipterans [38–44]. The Middle-Late Triassic was a period of extraordinary diversification of the Holometabola, which comprises most of the modern-day insect species, but also for various lineages and groups of hemimetabolous insects [45–47]. Moreover, the diversification of aquatic insects (a key event of the “Mesozoic Lacustrine Revolution”) had already begun by the Middle Triassic, providing new insights into the early evolution of freshwater ecosystems [46]. The epic rainy spell — known now as the Carnian Pluvial Episode (CPE) — took place from around 234 to 232 million years ago and marked a stark shift from the typically arid conditions of the Late Triassic [48]. There was a marked rise in rainfall (at least four episodes of increased rainfall have been deduced from sedimentary and palaeontological data; [48–51]. This led to extensive environmental changes and the subsequent demise and then collapse of many ecological systems. The shifts in climate encouraged substantial changes in global flora too. Many new types of plants emerged that were more suited to the humid climate. Several late-diverging fern families emerged and the Bennettitales (cycad-like plants), diversified. Extensive coal deposits formed once again, and it seems that the first substantial coal is produced since the Permian. Conifers seem to have benefitted and the CPE provides the first major finds of amber in the fossil record [52–54]. Major evolutionary innovations followed the CPE, and coincided with marked faunal changes in terrestrial and marine ecosystems, such as the first occurrence of dinosaurs, lepidosaurs, an expansion of coniferous trees, calcareous nanofossils and scleractinian corals [48,55–59]. The CPE was a period of ‘turnover’, effectively paving the way for the dominion of the dinosaurs and the evolution of many terrestrial animal groups that still roam the Earth today. Under such highly challenging circumstances and in the transforming, competing world, ancestral scale insects, similarly as aphids, became modified, probably due to the diminution of the body size and they probably became more cryptic, avoiding competition and potential enemies, but also using new host plants, new ecological niches in their roots, in humus and litter. Eventually the Coccinea diverged into numerous groups as early as the Late Triassic-Early Jurassic [19,28,60]. One of the morphological changes observed is the transformation of the leg into a digging organ. The coccid legs are well equipped for crawling directly on the ground, among and under soil particles, plant parts, humus, detritus, etc. This kind of locomotion in combination with swimming (“swimming” among solid particles) and digging does not require adhesive organs and a double claw to fix the position of the tarsus. The leg is fixed by sticking the apex of the tarsus into the soil; thus it should be short, sharp and tapered [19,61]. The hypogeic habitat of the archaic Coccinea could be a reason for the lack of fossils of this group in the Triassic and early Jurassic deposits. Male polymorphism is a peculiar feature of scale insects. They may be winged, brachypterous or apterous [16,19,62]. The supposed ancestral habitat of the archaic Coccinea exerted a strong pressure towards wing reduction, resulting in the loss of wings in females. The evolution of the male was directed by two stimuli: the habitat towards wing shedding, and reproduction, towards wing retention [19]. These are postulated to have resulted in the origin of four characteristic features of the coccid male. 1) The posterior wings are reduced either to halterae or completely atrophied – dipterization of flight apparatus, but with different biological background to that of aphids and flies. 2) The forewing is folded flat and overlapping along the abdomen in resting position, which facilitates the moving among soil particles, detritus, plants parts, etc. 3) The males are polymorphic with respect to the wings, i.e., all forms are affected by natural selection due to adaptations to different ecological conditions. 4) The males are dwarf-like to crawl among soil particles, and it is assumed that the evolutionary processes promoted this for bioenergetics reasons [13,19]. Thus, the males of archaic Coccinea became fragile, polymorphic gnats, when diminution of the body was probably realized by an early stopping of feeding. However, the emergence of male and female imagines must have been simultaneous, and therefore shortening of time of development in males was not possible. The solution “invented” by male Coccinea was the transformation of the last male larval instars into resting stages with enlarged wing buds (the third stage is non-feeding but mobile, while the fourth instar resembles the pupa in the Orthezioidea; third and fourth instars, i.e., prepupa and pupa, are immobile and non-feeding among Coccoidea). Subsequently the wing buds in the resting stages become enlarged, whereas in the female larvae reduced. The simplification of the male connected with the wing reduction is realized in two ways: with retention of resting stages (the rule) – this process may be understood as larvalization or with elimination of these instars, the process understood as neoteny. Scale insects probably lost the rhinaria, serving as distant chemoreceptors for searching for host plant location at a distance [63–65]. The processus terminalis of the antenna occurring in aphids was lost in Coccinea probably due to male aphagy and female apterism; the large number of flagellar joints in some coccids seems to be secondary [60]. The regressive transformation of the cephalothorax, antennae, legs, sense organs, etc., so the appearance of some wingless males is exactly the same as that of larva. Structural simplification, and a larviform body structure because of wing reduction and lowering of larval instars, larvalization seems to be rightly assumed also for females [13,19]. A diverse array of genetic systems exhibited in scale insects [13] further promotes conflicts of interest over transmission and sex ratio between male- and female-expressed genes, parental- and offspring-expressed genes (both examples of intra-genomic conflict) and between scale insects and their endosymbionts (inter-genomic conflict), and its origins seems to be with major evolutionary and biological change in scale insects behavior in the Triassic. An obligate symbiotic relationship with one or more species of bacteria is present in the vast majority of scale insects and aphids. Scale insects rely on their endosymbionts to synthesize and provide the essential amino acids and vitamins absent in their diet [66–69]. If endosymbiotic bacteria and their hosts remain associated for a long evolutionarily time, they can co-speciate or co-diverge together. In those cases, there is a phylogenetic congruence that can be traced back to a single infection event [70]. Ross et al. [71] presented a formal analysis that shows support for the hypothesis that coevolution between scale insect host and endosymbionts with a male killing phenotype could have led to the evolution of male haploid genetic systems. A recent study of endosymbionts in Ortheziidae, Matsucoccidae and Steingellidae [72] revealed presence of the bacteria *Wolbachia* and *Sodalis* in Ortheziidae, *Wolbachia* in Matsucoccidae, while in Steingellidae the bacteria *Sphingomonas,* which are transovarially transmitted from mother to progeny. Rosenblueth et al. [69] suggested that the recruitment of endosymbiotic bacteria took place 150–250 Ma ago, at the same time as the scale insect origination and its first radiation. However, it must be taken into consideration that biochemical properties of gymnosperms’ phloem from the Triassic times was different from phloem of angiosperms, the host plants for the modern archeococcids. The assumption for coevolution of recently identified endosymbiotic bacteria since the Triassic seems to be of limited value, as angiosperm revolution and shift to these plants as hosts probably changed also endosymbiotic relationships of scale insects.

***Jankotejacoccus* Szwedo, Azar et Sendi, gen. n.**

urn:lsid:zoobank.org:act:60EDC0A9-FFAA-48EF-89F6-3D4E37052389

**Type species: *Jankotejacoccus*** *libanogloria* **Szwedo, Azar et Sendi, sp. n., by present designation and monotypy.**

**Derivation of name: Generic name is given in honour of the late Professor Jan Koteja (1932-2004), eminent Polish specialist on modern and fossil coccids, combined with the generic name ‘*Coccus*’. Gender: masculine.**

**Diagnosis: Alate male. Body minute, less than 1 mm long. Antennae shorter than body, antennal bristles about 4 times exceeding diameter of antennomere, arranged in whorls; apical antennomere without apical bristles (?). Prescutum sclerotized, convex, elevated. Legs long, setose; tarsal segments distinctly tapering apicad, about ½ of tibia length, tarsal claw single, distinct, two clavate tarsal digituli present. Penial sheet conical, short.**

***Jankotejacoccus libanogloria*** **Szwedo, Azar et Sendi, sp. n. (Fig. 1 C, and E)**

urn:lsid:zoobank.org:act:B6A07A3F-0A31-4C33-AEC9-F6A0CA4D29FD

**Material: Holotype, alate male, SNM Z 40023; deposited in the Slovak National Museum, Natural History Museum in Bratislava.**

**Derivation of name: Specific epithet is a combination of the Latin words ‘gloria’ (for glory, magnificence, honour or splendour) and ‘Libanos’ (for Lebanon, where the specimen was collected).**

**Description: Body slender, ca. 0.86 mm long without wax tail. Compound eyes ca. 0.2 mm in diameter, with 9 facets, each 0.025mm in diameter visible; Antenna with 10 segments, scapus merely longer (0.05 mm) than pedicel (0.04 mm), pedicel about twice as long as wide, flagellomeres III and IV about as long as pedicel, about 4 times as long as wide; flagellomeres V (0.07 mm) and VI (0.08 mm) twice as long as preceding ones; flagellomeres VIII-IX (0.11, 0.10; 0.09 mm) slightly longer than flagellomeres V-VI; apical flagellomere about as long as flagellomere III (0.04 mm). Flagellomeres III-IX with distinct, protruding whorls of setae; setae ca. thrice as long (ca. 0.03 mm) as flagellomere diameter (ca. 0.01 mm). Praescutum (?) elevated, bulging, scutum declivous, scutellum convex. Forewing hyaline, oval, with wide base and alar lobe present 0.69 mm long, 0.22 mm wide; anterior margin with a few microsetae; widely rounded in apical margin. Stem Sc+R (‘subcostal ridge’) shifted from margin, not reaching forewing apex; pterostigma weak, slightly sclerotized; ‘rs’ [RP] and ‘afx’ [trace MP] absent; ‘cua’ long reaching almost the margin; ‘pfx’ [posterior flexing patch, remnant of CuP] visible; clavus with vein Pcu (?). Forewing surface wrinkled, but not strongly sculptured. Halter present, narrow, elongate (?) with one hooked seta (?). Legs slender, covered with sticking out setae. Tarsomeres not fused with tibia. Profemur 0.07 mm long 0.03 mm wide, with row of short setae; protibia 0.15 mm long, with rows of setae; setae about as long as tibia diameter; tibial spurs present; protarsus 0.05 mm long, with two capitate digituli (0.01 mm long), tarsal claw 0.01 mm long, without (?) teeth. Mesofemur 0.08 mm long, with rows of setae; mesotibia ca. 0.15 mm long with rows of setae; setae about as long as tibia diameter; tibial spurs present; protarsus 0.05 mm long, with rows of setae, with two capitate digituli (0.01 mm long), tarsal claw 0.01 mm long, without (?) teeth. Metalegs hardly visible, tarsi as in pro- and mesoleg. Abdomen elongate, penial sheath ca. 0.06 mm long, wax tufts ca. 0.28 mm long.**

**Remarks: The state of preservation is insufficient to observe all features traditionally used in descriptive taxonomy of male scale insects.**

**Age and occurrence: Upper Jurassic, Kimmeridgian, Lebanon.**

**Supplementary Note 3:** Analysis of the Jurassic Aintourine amber

Jurassic amber of Aintourine itself is transparent, homogenous, pale orange-pale yellow (some unstudied pieces are mainly red) and it appears empty. Its extractability in solvent reached 59.9%. The total amber extracts (**Fig. 4B1**) contained sesquiterpenoids, diterpenoids, aliphatic hydrocarbons, and fatty acids. The major components of the extracts were abietane derivatives (**Fig. 1, and Supplementary Table 1**), including the most abundant compound 16,17,18-trisnorabieta-8,11,13-triene. In addition, a small amount of phenolic diterpenoid totarol was detected. In small amounts, *n*-alkanes (*n*-C_20-31_) and pristane were detected. Analysis of derivatized extract (Fig. 4B2) showed similar record as the underivatized extract. Only esters of fatty acids (lauric, palmitic, 9-octadecanoic), and other oxygen-containing compounds were also found. Using Py-GC/MS (**Fig. 4C12**), the dominant compound in the pyrolysate was ionene. Other identified compounds were abietanes and alkyltetralines. The TMAH-Py-GC/MS method (**Fig. 2B**) revealed similar results in the compound composition supplemented by fatty acid esters (**Supplementary Table 2**). However, 16,17-dinorcallitrisic acid methyl ester was detected in low concentration (see below).

Compelling evidence was found for compounds with abietane-type structures in the sample extracts. Abietanes are the largest class of tricyclic diterpenoids that have been described in all current conifer families [73]. Non-phenolic abietanes - structural products of aromatization and oxidation processes, were dominant in the extracts. 16,17,19-trisnorabieta-8,11,13-triene, a major compound identified in extracts could be diagenetic products of abietane or pimarane diterpenoids. Low quantities of pimaradiene were also identified. However, non-phenolic abietanes are widely distributed in all conifer families except Phyllocladaceae and due to the widespread distribution chemotaxonomical interpretation is hard. The aromatic derivatives may be products of deposition processes that had been preceeded in the in soil and sediment environments, mainly under aerobic conditions. The lack of phytane and presence of pristane in the extracts is evidence for this. The compound totarol is a member of phenolic abietane group that was found in extracts, and even at low concentrations considered as a chemotaxonomic marker for living Cupressaceae and Podocarpaceae. The compound 16,17-dinorcallitrisic acid methyl ester is usually reported to be a thermal product of Pinaceae and callitrisates in modern conifer resins is limited to the Cupressaceae [74]. Although phenolic abietane (totarol), a common constituent of Cupressaceae resins was detected, other typical compounds (cuparenes) were not found, with a possibility of an alternative source of callitrisates. The compound rimuene is another tricyclic diterpenoid identified in the extracts, which is a component of current resins found in plants of Cupressaceae, Taxodiaceae, Podocarpaceae and Phyllocladaceae.

The chemical analysis using Py-GC/MS showed the dominant compound was ionene. This compound might be a diagenetic product from labdanes, evidencing intensive degradation processes, which makes it hard to recognize parent structures. Among other products observed, abietane-type diterpenoids dominated. The sample is Class Ib amber according Anderson et al. [75] classification. The possible resin source among living families could be Cupressaceae or Podocarpaceae. Howevewr, intensive diagenetic and other processes might lead to failure to detect products that could indicate a different plant family. There might not be a modern analogue for the studied sample that would allow determination of the chemical biomarkers specific to this family. The chromatogram is by composition close to the record of Lower–Middle Cretaceous samples from Spain performed by Menor-Salván et al. [76]. They suggested as the possible botanical origin a member of the extinct Cheirolepidiaceae (259.0 to 61.7 Ma) which were coniferous plants similar to Cupressaceae. Their result was supported by leaves of the genus *Frenelopsis* (Cheirolepidiaceae) as the dominant macro-botanical remains in the amber beds. The macro-fossil record of the English Isle of Wight, southern England deposits also dominated by the extinct conifer family Cheirolepidiaceae, and Py-GC/MS analyses of amber indicate a terpene distribution dominated by abietane- and labdane-type terpenes. These ambers were assigned to either the Pinaceae (on the basis of negative evidence of markers) or to the Cheirolepidiaceae [2] supported by macrofossils. The cheirolepidiacean origin is additionally supported with amber producing tree identified here as *Protopodocarpoxylon*, with its leaves identified here as *Brachyphyllum* (both preserved in contact with amber) and pollen aff. *Classopolis.*

**Supplementary Notes 4:** Organic petrography of the amber-bearing sediment and amber

3.1 Optical characteristics of amber

A few semi-oval and angular inclusions of colorless transparent amber firmly connected to fine grained sediment were studied microscopically under UV and white reflected light. The boundaries between amber and sediment are either sharp (**Supplementary Fig. 1**) or have the character of injection or embossing (**Supplementary Fig. 2.2, 3**). In some cases, there are rod-shaped or disc-shaped structures on the edge of the individual pieces of amber which have a reddish-brown color under UV light (**Supplementary Fig. 1.4-6**).

Under UV light excitation, amber exhibits white to greenish-blue color and intense opalescence. Decreased intensity of fluorescence and dull yellow color is observed in some small isolated pieces of amber drowned in sediment (**Supplementary Fig. 1.1, 2**). One unspecified fungal ascospore (**Supplementary Fig. 1.7**) besides a few fragments of plant tissues of undetermined origin are embedded in the amber (**Supplementary Fig. 1.8**).

Amber is dark wine-red under white incident light. Darker varieties are typical mainly for some isolated pieces.

3.2 Optical characteristics of organic matter in sediment

A large amount of organic matter of terrigenous origin is found in the sediment associated with amber. Microscopic analysis of organic particles termed “macerals” was performed in both incident white and UV light. Macerals of huminite and liptinite group, as well as char particles, have a dominant representation in the sediment.

Macerals of huminite group are derived from the tissues of roots, stems, barks, and leaves composed of lignin, cellulose, partly also from tannins [77]. The most abundant macerals of this group are textinite and in lesser amounts ulminite. Under the white light, the presence of both darker and lighter varieties of these macerals (textinite A, B and ulminite A, B) are confirmed). They show dull yellow to reddish-brown and weak dark brown fluorescence under UV-light excitation. Textinite cell lumens are open or filled with corpohuminite and resinite. Corpohuminite (**Supplementary Fig. 2.9, 11),** and its type phlobaphinite (**Supplementary Fig. 2.10**) are found in relatively large amounts either as isolated bodies or together with the textinite, attrinite and macerals of the liptinite group. Both macerals have grey color in white light and don´t fluoresce under UV light.

Some oxidized or recycled vitrinite particles are present in sediment. In white light, they have a paler gray color than huminite macerals and do not show any color after excitation by UV light.

Liptinite macerals consist predominantly of bituminite III, cutinite, resinite, liptodetrinite and minor alginite and sporinite. Bituminite III occurs in the form of lenses of irregular shapes and as “matrix bituminite” which is merging with the groundmass and visible only under UV light excitation. All forms show a brown-reddish florescence (**Supplementary Fig. 2.12**). Dark yellow fluoresce pore fillings in char particles indicates the presence of bituminite as well (**Supplementary Fig. 2.13**).

Cutinite (**Supplementary Fig. 2.14-16**) is present in its most typical form in strips with serrated edges. Most often, strips of cutinite are found in pairs in which the serrated edges face each other (**Supplementary Fig. 2.15**) indicating a depositional environment without bioturbation. In fluorescence mode, cutinite is golden yellow to orange and dark grey in white light.

Studied sediment contains relatively higher amount of suberinite**.** Suberinitic tissue is gold to –dirty yellow under UV light and dark grey under white light. Most often the cells are empty and compressed (**Supplementary Fig. 2.11**). In some cases, the suberinite occurs together with phlobaphinite, which fills the cells (**Supplementary Fig. 2.10**). Suberinite and cutinite show wide range of preservation stages from well preserved to highly degraded and fragmented.

Resinite occurs in the form of oval bodies as in situ cell fillings in textinite **(Supplementary Fig. 2.17**), but it is also found in a form of smudges and diffuse impregnation. In the reflected white light resinite is dark grey. Under UV excitation is white-yellowish or light yellow-greenish (fluorinite- a variety of resinite).

Alginite and sporinite are present in lesser amount. Because of advanced microbial degradation, more precise distinguishing from the other liptinite macerals is difficult. Besides, the vast majority of liptinite macerals have very similar color and intensity in fluorescence mode. Lighter fluorescent color and slightly higher fluorescent intensity enable identification of sporinite (**Supplementary Fig. 2. 18**). A few individuals resembling *Botryococcus braunii* colonial alga are present in the sediment (**Supplementary Fig. 2.19**).

Liptodetrinite contributes remarkably to the total volume of organic matter in the sediment. It is almost invisible in white light, and in UV mode, it has a golden yellow color, almost identical to cutinite and suberinite. It is likely that a large portion of liptodetrinite mostly consists of mechanically and microbially degraded cutinite and suberinite.

Abundant char particles found in the studied sediment are characterized by white color under incident white light, very high reflectivity and by randomly distributed pores (**Supplementary Fig. 2.20**). A vast prevalence of them are chars with high percentage of unfused material with irregular and angular shapes. The most abundant char particles with solid/ fusinoid morphologies have very little or no porosity in contrast to less numerous inertoid forms, which have a higher porosity.

The highest porosity has particles with one large central void (primary porosity). These char type could be classed as a sphere [28]. A lesser amount of thick-walled particles with a few similar sized pores is classed as a network char type.

3.3 EPMA and XRD analyses

Sedimentary groundmass of the amber holotype (Fig. 3C) predominantly consists of a massive (cryptocrystalline to fine-grained) aggregate of böhmite [γ-AlO(OH)] with a minor admixture of kaolinite, disseminated, and probably clastic fragments of heavy minerals: abundant pseudorutile, rare anatase, rutile, zircon and monazite-(Ce), tiny irregular veinlets of goethite as well as an unidentified aluminium phosphate-sulfate mineral (Fig. 3C). Böhmite shows a nearly end-member composition with small admixture of Si, Ti and Fe (Supplementary Table 1). Pseudorutile forms euhedral to subhedral pseudomorphs after primary ilmenite, usually ~15 to 70 μm across (Fig. 3C). Their chemical composition (Supplementary Table 2) is analogous to hydroxylian pseudorutile with a composition between Fe^3+^_2_Ti_3_O_9_ and Fe^3+^Ti_3_O_6_(OH)_3_ [22,23] probably with a submicroscopic admixture of secondary rutile and/or anatase (TiO_2_), SiO_2_ and AlO(OH) compounds. Conversely, slightly elevated contents of MgO (1.2-1.5 wt.%), MnO (0.4-0.6 wt.%) and V_2_O_3_ (0.2-0.4 wt.%) indicate isomorphic admixtures of primary ilmenite, accommodated into pseudorutile structure.

**Supplementary Notes 5:** Reflectance Analysis

The reflectance measurements were carried out on the ulminite, textinite, corpocollinite- phlobaphinite, vitrinite and char (**Fig4e, and Supplementary Fig. 3**). Mean random reflectance of macerals are given in **Supplementary Table 1**.

The reflectance of the A varieties of textinite and ulminite (0.38 %Ro) is lower than reflectance of ulminite B (0.4 %Ro). The reflectance of corpohuminite (0.42 %Ro) is slightly higher than ulminite B. Mean reflectance of char particles is 4.68 %Ro.

**Supplementary Notes 6:** Discussion on thermal maturity and characteristics of depositional environment

**Thermal maturity**

Comparing the maturity of fossilized resins with accompanying sediment can provide information on the maturity, relative age, and history of amber alteration and taphonomy.

The increasing depth of burial leads to an increase in temperature, which together with an increasing heating time leads to accelerated maturation of the organic matter in the sediment as well as the rise of coal rank. The reflectance of ulminite B is used as a rank parameter [77]. The mean random reflectance of ulminite B 0.4% Ro means that the coalification of the humic particles present in the amber-bearing sediment corresponds to a low rank coal at the boundary between brown coal B and sub-bituminous coal A (light brown coal A). Based on reflectance data, the maximum temperature of amber-bearing sediment probably did not reach even 40° C [78] during its geological history.

On the other hand, the high reflectance of the char particles indicates the high temperature to which the organic matter was exposed during a relatively short wildfire in the past. Natural char is formed by the influence of heat from the fire on coal or gellified organic matter peat [79] so its presence in sediment indicates paleopeat fire (probably ground fire).

The nature of the coalified or peatified organic matter, amount of oxygen, the temperature and duration of combustion all play a role in determining the morphologies of char [79–81] and their reflectance [82–84]. Based on various studies [82,83,85,88], and the analyzed average reflectance of char particles 4.68% Ro, the temperature caused by the peat fire could correspond to 595–800 °C. Amber, as a fossilized resin (resinite) is sensitive to ambient temperature [89–92], but, as for the reflectance, it reacts differently than ulminite or other organic particles. At the same thermal conditions, amber always has a lower reflectance than ulminite B.

The reflectance of amber/resinite is also influenced by sunlight, as due to photochemical reactions, changes occur in the amber leading to an increase in reflectance [92]. Photo-degradation can actually cause significantly greater changes in amber structure than thermal degradation (at temperatures up to 85 °C). Similarly, the effect of oxygen, moisture and salt influence the maturity and alteration of amber [93–94]. Oxidative degradation of amber can occur within a few years, particularly in exposure to ultraviolet light [29].

Analysis of amber confirmed very low reflectance, which can indicate that the thermal history of amber and sediment was simultaneous. Moreover, most studied amber pieces have the same reflectance, and fluorescence color and intensity, which means that the resin, before it got into the sediment, was not exposed to sunlight and photo-oxidation, or influenced by heat from the fire and probably get into the sediment in a relatively fresh state.

However, some isolated pieces of amber with lower intensity of fluorescence, dull yellow color (**Supplementary Fig. 1.1**) and slightly increased reflectance may indicate some degree of alteration caused by the above-mentioned effects.

**Characteristics of depositional environment**

Maceral analysis is used for characterization of the depositional environment of sediment and organic matter (e.g., [95–98] and it is also a good tool for interpretations of amber taphonomy [92,99,100].

Qualitative maceral study showed a presence of organic matter derived from higher land plants in the amber-bearing sediment. A presence of marine-derived alginite was not sufficiently demonstrated due to relatively advanced mechanical and microbial degradation of organic material.

Terrigenous macerals present in sediment originates mainly from both herbaceous and arborescent higher plants. According to Sýkorová et al. [101], Pickel et al. [102] and Petersen [79], individual parts of these plants represent the following macerals that have been identified in the sediment: a) woody tissues and the cellular contents of roots, stems, barks and leaves (textinite, ulminite, corpohuminite and phlobaphinite); b) plant resins, copals and waxes, which occur as cell excretions in different parts of plants like leaves, wood, and bark (resinite); c) fossil cuticles, which form protective layers of leaves and stems (cutinite); d) periderm cells, which usually cover the stalks, branches, stems roots, fruits and bulbs (suberinite), e) plant spores and pollen (sporinite); f) *Botryococcus*-type algae derived from phytoplanktonic fresh and brackish water green algae (alginite); g) amorphous material which is a degradation product of mainly humic origin formed in a water environment with reduced oxygen supply (bituminite III); h) highly reflecting particles of natural char formed by the influence of heat from wild fire on gelified organic matter in a peat; and, i) recycled or oxidized vitrinite (recycled coalified woody tissue derived from the eroded older sedimentary rocks or from dried and oxidized parts of peat).

Association of above-mentioned macerals could indicate depositional environment of amber bearing sediment, which could represent a place in close vicinity of forested swamp peatland, or peatland edge forest where fine-grained sediment or soil was accumulated together with litter from vegetation. High proportion of bituminite III represents degradation product of various liptinitic precursors formed in water environment under reducing conditions [102]. On the other hand, increased proportion of textinite at the expense of ulminite can indicate a higher degree of cell tissue preservation under relatively dry periods and lower pH [96]. The presence of large amounts of maceral cutinite originating mainly from the cuticles of leaves may suggest that the fallen leaves formed a layer covering the ground surface at the site where the resin fell from the resin-producing tree. This is also evidenced by several finds of amber pieces, which are partially or completely wrapped in leaves (cutinite), (**Supplementary Fig. 2.14, 16**).

Suberinite as well as corpohuminite, are relatively resistant to microbial decomposition, so it is possible that increased abundance of these macerals can be a result of concentration in a consequence of selective preservation [103]. Large number of char particles was formed during the fire of peat located near the site of amber and sediment deposition, where they could be blown by the wind.

In general, the presence of alginite can be considered as evidence of sedimentation in an aqueous environment. Unicellular and filamentous alginite was not found. However, it cannot be ruled out that alginite is present as liptodetrinite or in the mineral-bituminous groundmass.

A small number of colonial microalga *Botryococcus braunii* in the sediment suggest lacustrine environment.

Generally, high pyrite contents are associated with marine-influenced sedimentary organic-rich sedimentary deposits [104]. Since no pyrite was detected under optical microscopy, it can be assumed that the site where the amber was found was isolated from marine waters.

**Summary**

The amber and amber bearing sediment was studied under reflected-light microscopy. The boundaries between amber and rock have the shape of straight lines or curved contours. Narrow and sharp edges suggest that brittle (broken) resin got into sediment, while the semi-angular edges suggest the penetration of a low-viscosity resin into empty or partly waterlogged cavities. Low viscosity can partially explains the absence of insect inclusions within amber. In some cases, there are rod-shaped or disc-shaped structures on the edge of the individual pieces of amber that have a reddish-brown color under UV light. These shapes probably formed when the low viscosity resin fell into the aqueous environment. Several pieces of amber are entirely wrapped in a few layers of orange fluorescing cutinite (fossil cuticles derived from leaves), suggesting that the resin fell from the tree on the earth surface covered by leaves. Amber is usually clear, some inclusions of fungal ascospore, mineral and organic debris (char, leaves, woods, and fungal tissues) are rarely found in the marginal parts of amber. There are no indications of longer transport and re-sedimentation of the amber, so the depositional environment was probably located close to the resin-bearing parent tree. The amber-bearing sediment contains organic residues originating mainly from higher plants as well as fossilized sapropel and a very small amount of algae, indicating an occasionally waterlogged environment near the forest, forested swamp peatland, or peatland edge forest. Here, litter from vegetation was deposited together with acidic fine-grained lateritic soil developed by intensive and prolonged weathering of the underlying Kimmeridgian basalts. Over time, a peat swamp developed at this site and a part of the organic debris mixed with the soil underwent microbial decomposition in a low oxygen aqueous environment. This is supported by the presence of a higher quantity of fossilized sapropel (bituminite-III), which was formed under reducing conditions by microbial degradation of organic debris. Increased amounts of certain specific parts of vascular plants, such as e.g. bark of branches and roots, leaf cuticles and resins (macerals suberinite, cutinite, and resinite) may be the result of concentration due to selective preservation, as these are more resistant to microbial degradation. Since no pyrite was detected, it can be assumed that the site where the amber was found was isolated from marine water during the time of deposition of the analyzed sediment. Based on mean reflectance of maceral ulminite B (0.4 %Ro, **Fig. 4e**) the maximum burial temperature of amber and amber-bearing sediment probably did not reach even 40 °C during whole their geological history. Windblown heat-modified char particles found in the sediment suggest ground paleopeat fire that occurred at a distance from the amber deposition site. The temperature in peat during a fire could have reached 595 to 800 °C.

**Supplementary Notes 7:** IR spectroscopy

In general infrared spectra of ambers composed from polymeric molecules mostly display absorption bands at similar wavenumbers, however their relative intensities differ because quantitative contribution of specific functional groups vary according to the volume of particular molecular components. Therefore ambers from various deposits usually provide distinct shapes of their spectra which reflect various factors, such as maturation, origin and also environment they occur [105–110].

**Aintourine amber – band assignment**

Spectra of ambers from Aintourine were measured form three pieces that slightly differed in colour. Cretaceous Lebanese amber of Hammana/Mdeyrij of different origin is also compared. Spectra were analysed in two regions separately, 4000-2500 cm^-1^ and 1900-400 cm^-1^ (**Supplementary Figs. 4, 5)**. The first region gives information on stretching modes of O-H, C- H vibrations, therefore this region is also well suited to recognise bands attributed to admixtures that can occur in ambers. High wavenumbers bands at 3693 and 3622 cm^-1^ and 3530 cm^-1^ do not belong to vibrations from C-H bonds, therefore they could tentatively be attributed to the vibrations of admixtures containing hydroxyl groups in their structure and were detected also using other methods in sedimentary matrix. The first two bands most probably belongs to structural OH groups of kaolinite admixture as the position of the bands are typical for this clay mineral and they can be detected in the spectra even at very low kaolinite content [111]. Band near 3530 cm^-1^ could belong to the vibration of OH groups from inorganic admixtures containing aluminium and/or iron within their structure [112]. The assignment of the band could be tentative as OH groups from several minerals could give absorption bands in this spectral region. The above bands are visible as small components stuck to a complex broad band that spreads within 3700-3200 cm^-1^ that is attributed to absorption bands of OH groups from alcohols present in resins and also from water molecules. The shape of the OH band and its intensity varied between three species of Aintourine amber reflecting thus various amount of hydroxyl groups. The most intensive bands in the spectra of ambers belong to stretching C-H vibrations and occur between 3100-2700 cm^-1^. The assignment of the bands at 2931 cm^-1^ and 2846 cm^-1^ is unambiguous; these originate from symmetric and asymmetric vibrations of methylene groups (ν_a_ and ν_s_). Methyl groups provide bands visible in the spectra as obscure shoulder near 2957 cm^-1^ as ν_a_, while ν_a_(CH3-) is present downwards at lower wavenumbers at 2871 cm^-1^. High intensities of these bands reflect a dominance of aliphatic/alicyclic methyl and methylene functional groups as the most abundant molecular constituent in the amber samples. The small peak near 3077 cm^-1^ could indicate presence of C-H groups attached to aromatic rings, while peaks near 2731 cm^-1^ belong to C-H from functional group of aldehydes. Considering shape of C-H vibrations all three samples of Aintourine amber are identical. Exception is visibility of the band C-H from aromatic ring that is most visible in spectrum marked as ‘a’ (**Supplementary Fig. 4**). Further in the spectra another intensive band is present at 1699 cm^-1^ and is accompanied by well recognisable shoulder near 1739 cm^-1^. In overall, band is characteristic for vibrations of C=O groups. While sharp peak represents vibrations from C=O of carboxylic acids, shoulder to carbonyl groups from esters. The inflection near 1780 cm^−1^ was attributed to conjugated anhydrides [113]. At lower wavenumber side, a small feature near 1610 cm^-1^ corresponds to C=C vibrations aris–ing from the aromatic rings. Bending mode δ of methyl and methylene is visible as two component peak at 1464 and 1448 cm^-1^, respectively. Symmetric δ_s_CH_3_ is present near 1376 cm^-1^. Below this wavenumbers several bands can be detected. Their exact assignment is complicated and usually overall shape of bands is taken as indication of distinctions or similarities of ambers. The bands are connected with the features observed in previously described regions. Therefore, bands between 1300-1200 cm^-1^ were attributed to the C-O single bond stretching mode due to presence of carboxylic acids [113,114] however overlapping of various bending modes of C-H bonds was also mentioned in fossil resins samples [113]. For amber recognition the band near 1157 cm^-1^ appears to be interesting as it is connected with the C-O stretching bond of esters [106]. The high content of esters in Baltic ambers was reflected in its sharp shape which tower up over other bands in this region and resulted in the characteristic shape of this band in the ambers of Baltic origin. Bands near 1031 and 974 cm^-1^ belong to C-O stretching vibration, for example from alcohols [115]. From the point of amber maturation, band at 887 cm^-1^ is interesting as according this band the ambers maturation can be assessed [110,113]. Band belongs to exocyclic methylene groups, e.g. and its assignment is conditioned by the presence of other bands near 3078 and 1645 cm^-1^. Unless all bands are detectable in the spectra the assignment is not clear.

Aintourine samples show below wavenumbers 1900 cm^-1^ more pronounced differences in the spectra in comparison to region 4000–2600 cm^-1^. For example, the intensity of the C=O band near 1700 cm^-1^ shows variations relative to dominant band of νC-H and to δC-H (**Fig. 4D and Supplementary Fig. 4.1**). The highest intensity of C=O band is shown spectrum a compared to spectra c, b. Further alteration in the spectral shapes within spectral region 1300-900 cm^-1^ could reflect the number of carboxyl vs. ester and or ketone groups. The higher intensity of C=O bands results in more pronounced intensity of C-O single bond bands. Spectrum c also display the highest intensity of the 889 cm^-1^ band, while for other samples is scarcely visible (**Supplementary Fig. 4.1**). In this case the presence of other two corresponding bands near 3070 cm^-1^ and development of the shape of the spectra near 1640 cm^-1^ could be indication of the exomethylene groups. Despite consistency in the presence and position of the majority of the bands, even sample from the same deposit show some differences although general features are maintained.

**Comparison of Jurassic ambers from Lebanon: Aintourine (this work) with other Jurrasic ambers from reports [116]**

Spectra of Aintourine ambers were compared with the other Lebanese Jurassic ambers reported. Nine localities with Jurassic ambers were studied by Nohra et al. [116]. To assume similarities between other Jurassic ambers studied in this work with already reported ambers, the intensity of C=O band relative to δC-H was considered. The higher intensities C=O displayed El-Ghineh, Ehmej-Aalmat, Arz Tannourine and Beit Mounzer. In addition, the shapes of the spectra below 1300 cm^-1^ more less follow the shape and intensities detected for Aintourine. In the work of Nohra et al. [117], with the same approach, *i.e.,* by comparison of the intensities of the C=O bands. IR spectra of Aintourine presented in their work is partially similar to the spectra from this study (**Fig. 4D, and Supplementary Fig. 4, 5**). Small distinctions in the spectral region 3600–3200 cm^-1^ and below 1000 cm^-1^ could reflect the admixtures remaining in the bulk samples of amber. Also shape of spectra within 1200–1300 cm^-1^ do not completely copy the shape of spectra for the same amber. However absorption bands in this region were slightly altered even for three representative samples introduced in the (**Fig. 4D, and Supplementary Fig. 4a**–**c**). A similar development of the spectra in finger print region could be given to the spectra of Haouqa II [116]. Besides ambers from Jurassic authors also included one Cretaceous amber sample from Hammana/Mdeyrij for comparison, that is also introduced (**Fig. 4D, and Supplementary Fig. 4d**). Besides lower resolution of νC-H in region 3100-2700 cm^-1^, the spectra in [116] are almost identical with those presented in **Supplementary Fig. 4.** When comparing spectra of Jurassic Aintourine with Cretaceous amber Hammana/ Mdeyrij there are quite a lot of differences. The most characteristic could be: lower intensity of C=O groups characteristic bands relative to bending vibrations of C-H groups, adverse contribution of carboxyl (1700 cm^-1^) vs. ester groups (near 1740 cm^-1^), subsequently more pronounced intensities of two bands attributed to alcohols near 1030 and 973 cm^-1^. The low intensity of 887 cm^-1^ band indicate minor contribution of exomethylene group, while bands near 850 and 812 cm^-1^ show higher intensities compare to Aintourine.

**Comparison of ambers Aintourine with selected European ambers (this work)**

From selected cheirolepidiacean Cretaceous types of amber samples, only spectra of French Fouras strongly resemble Jurassic amber from Lebanon (**Supplementary Figure 5A**). As provided in [117] Fouras give heterogeneous spectra showing both, either similar relative intensities νC-H vs. νC=O with ratio close to 1, but also considerably lower ratio was detected for the other spectrum. The spectra of other European ambers selected for this study, i.e. San Just from Spain and SVK amber from Nemšová (**Supplementary Figure 5B**) have strong affinities with the spectra of Hammana/Mdeyrij amber (**Supplementary Figure 5B**). Those ambers even contain the same type of admixtures represented by the bands near 3635 and 3530 cm^-1^. San Just amber is sample with spectra already reported in other works e.g. [3], while spectra measured for SVK amber from Nemšová are reported for the first time. As indicated by the pronounced intensity of the bands around 3440 and 1027 cm^-1^ San Just is abundant on OH groups from alcohols. Isle of Wight amber (**Supplementary Figure 5C**) in comparison produced spectra with higher intensity of C=O carboxyl bands on expense of C=O from esters (near 1723 cm^-1^), therefore trend similar as distinguished for Aintourine and Fouras amber, however the overall intensity was lower relative to νC-H bands at 2928 cm^-1^ compared to intensity in Aintourine (**Supplementary Figure 5A**). The rest of the spectrum looks like those obtained for other Cretaceous ambers from this work. There is strong divergence in the region between wavenumbers 1640-1600 cm^-1^ for spectra reported in [118] showing strong band in this part of spectra and for sample introduced in **Supplementary Fig. 5C** that did not provided significant feature. Bands attributed to admixtures also correspond to those observed in Aintourine amber providing bands near 3699, 3630–3620 and 3530 cm^-1^. In addition, Isle of Wight sample provided for IR measurement, display more pronounced intensity compared to other studied ambers showing clear bands in the high wavenumber region and also below 600 cm^-1^.

**Supplementary Notes 8: analysis of characters distribution and character states of *Jankotejacoccus***

**Parsimony analysis was performed to reveal distribution of characters available and compare the new fossil with the closest chronophyletical fossils. The set of characters available from the new fossil, Cretaceous archeococcids and selected living taxa was composed in the filedata matrix and analysed. The nexus data file was prepared with Mesquite 3.91 build 955 [119]. Trees were calculated with have been calculated with TNT 1.6 [120] and analysed with ASADO 1.85 [121], Traditional Search (TS) and New Technology Search (NTS) were performed with Equal Weighting (EW) and Implied Weighting (IW) [121,122], with different values of k parameter [123]. The most parsimonious trees with the same topology, were obtained from both analyses, with Implied Weighting with k parameter 12 for fossils and missing data treatments. The characters are checked under three different constraining criteria: unambiguous character changes, ACCTRAN (accelerated transformation; optimized for the earliest possible changes given ambiguity), and DELTRAN (delayed transformation; optimized for the latest possible changes given ambiguity), according to discussions presented by Agnarsson and Miller [124]. As results, the most parsimonious trees with the same topology, have been received in TS and NTS analyzes, with IW k parameter 12 [123] for fossils and missing data treatments.**

1. Number of antennal segments. 0= of aphid type; 1=less than 10 segments; 2=10 segments
2. Relative length of antennal setae. 1=longer than antenna diameter; 2=shorter than antenna diameter
3. Fleshy setae on antennae. 0=present; 1=absent
4. Bristles on antennae. 0=present; 1=absent
5. Antennal capitate setae. 0=absent; 1=present
6. Flagellomere structure. 0=filiform; 1=nodose
7. Reticulations on antennae. 0=absent; 1=present on pedicel; 2=on other antennal segments
8. Eyes. 0=compound; 1=simple
9. Number of ommatidia or individual eyes. 0=between 10 and 50; 1=between 50 and 100; 2=more than 100; 3=seven pairs of eyes; 4=four pairs of eyes
10. True ocelli. 0=absent; 1=present
11. Functional mouthparts. 0=present; 1=absent
12. Prothorax. 0=sclerotized; 1=membranous
13. Wing venation other than the anterior wing ridge. 0=cubital ridge only; 1=cubital ridge and other veins
14. Subcostal ridge of forewing. 0=extending to the tip of the wing; 1=extending to less than 3/4 the anterior margin
15. Pterostigma. 0=absent; 1=present
16. Ratio of wing to antenna. 0=wing longer than antenna; 1=antenna longer than wing; 2=subequal
17. Base of the wing. 0=narrow; 1=broad
18. Apex of the wing. 0=round; 1=pointed
19. Wings microtrichia. 0=absent; 1=present
20. Hindwing. 0=present and completely developed; 1=modified to hamulohalteres; 2=reduced but not hamulohalteres
21. Shape of the hindwing. 0=narrow and long; 1=broad; 2=intermediate; 3=not hamulohalteres.
22. Number of hamuli. 0=absent; 1=one; 2=two or more
23. Location of the hamuli. 0=on the anterior ridge of the hindwing; 1=at the tip of the hindwing.
24. Number of claw digitules. 0=two; 1=more than two; 2=absent
25. Number of tarsal segments. 0=one tarsal segment; 1=two tarsal segments with the one between tibia and tarsal segment 2 reduced; 2=at least two well-developed tarsal segments
26. Type of claw digitules. 0=clavate; 1=absent
27. Tarsal digitules. 0=absent or hair-like (i.e. undifferentiated); 1=present as one pair of thinly clavate digitules; 2=present as more than one pair of thinly clavate digitules
28. Number of claws. 0=one; 1=two
29. Claw denticle. 0=absent; 1=present
30. Type of tibial spurs. 0=spinose; 1=clavate; 2=undifferentiated
31. Abdominal caudal extension location. 0=abdominal segment VIII only; 1=absent
32. Wax filament secretion on at least one abdominal segment. 0=absent; 1=present
33. Penial sheath. 0=triangular; 1=spine like
34. Relative size of the penial sheath (length divided by base width). 0=between 0 and 1; 1=between 1 and 5; 2=between 5 and 10; 3=more than 10
35. Endophallus. 0=present; 1=absent

**Supplementary figures**

***
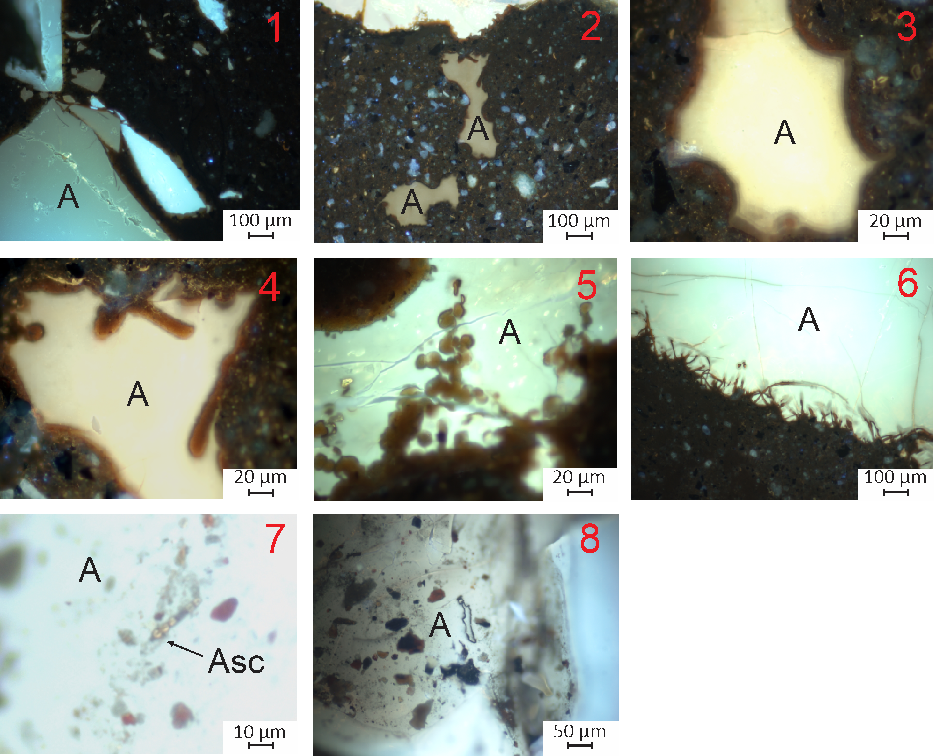
***

**Supplementary Figure 1.** 1-8 Aintourine amber under incident UV light. (1) Sharp amber-sediment boundaries. (2, 3) Injection and embossing-type structures in amber-sediment boundaries. (4, 5, 6) Rod, and disc-shaped structures on the edge of amber. (7-8) Inclusions of various origin embedded in the amber. (7) Fungal ascospore (8) Fragments of plant tissues.

***
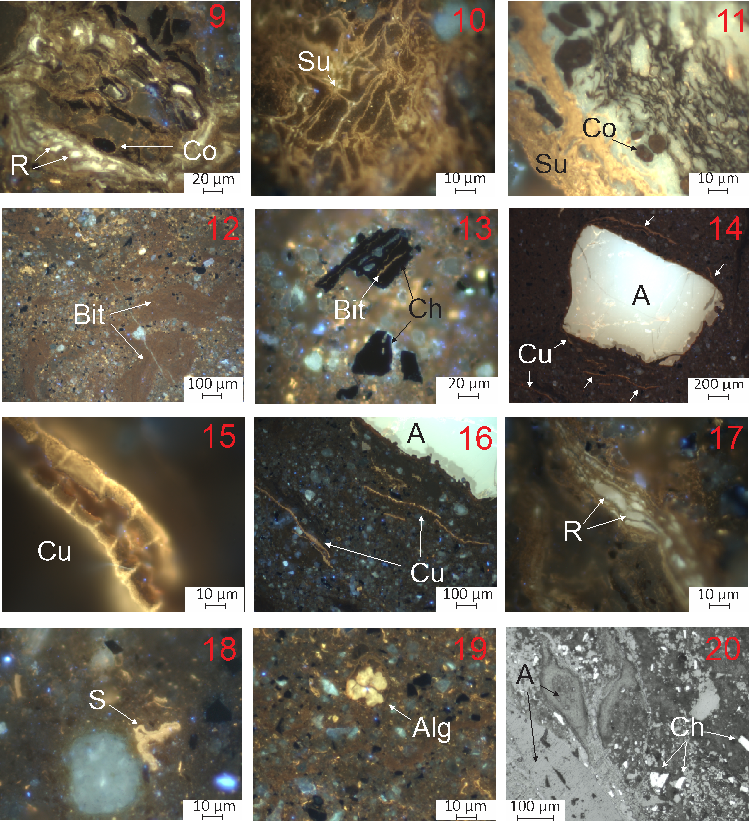
***

**Supplementary Figure 2.** 9-20 Organic matter in Aintourine amber-bearing sediment. (9) Dark non-fluoresce corpohuminite bodies (Co) and white-yellow resinite corpuscles (R). (10) Brown non-fluoresce corpuscles of phlobaphinite (pseudo-phlobaphinite) in yellow fluoresce suberinite tissue (Su). (11) Dark corpohuminite bodies (Co) and gold yellow suberinite (Su). (12) Brown-reddish fluoresce bituminite (Bit) and gold yellow fluoresce liptodetrinite. (13) Dark-yellow fluoresce filling cracks and pores (Bit) in non-fluoresce char (Ch). (14) A piece of amber (A) completely wrapped in a few layers of orange fluoresce cutinite - Cu (fossil cuticles derived from leaves). (15) Cutinite (Cu) – upper and lower part of leaf cuticle. (16) Cutinite (Cu) and amber (A). (17) White-yellow resinite corpuscles (R). (18) Sporinite (S). (19) - Tiny yellow liptodetrinite particles and alginite (Alg) that resemble the external shape of green colonial *Botryococcus braunii* alga. (20) White angular char particles (Ch) in sediment. Microphotographs (9-19) UV light excitation, and (20) white incident light.


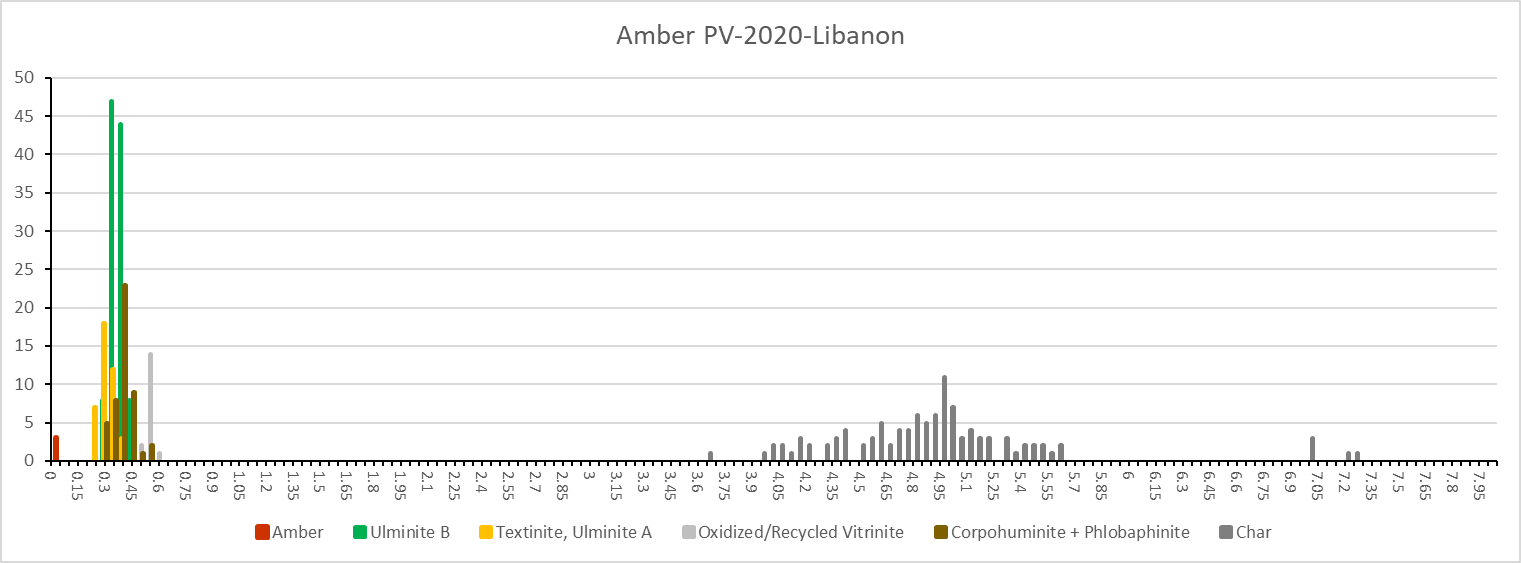


**Supplementary Figure 3** Reflectance histogram of **(21)**.


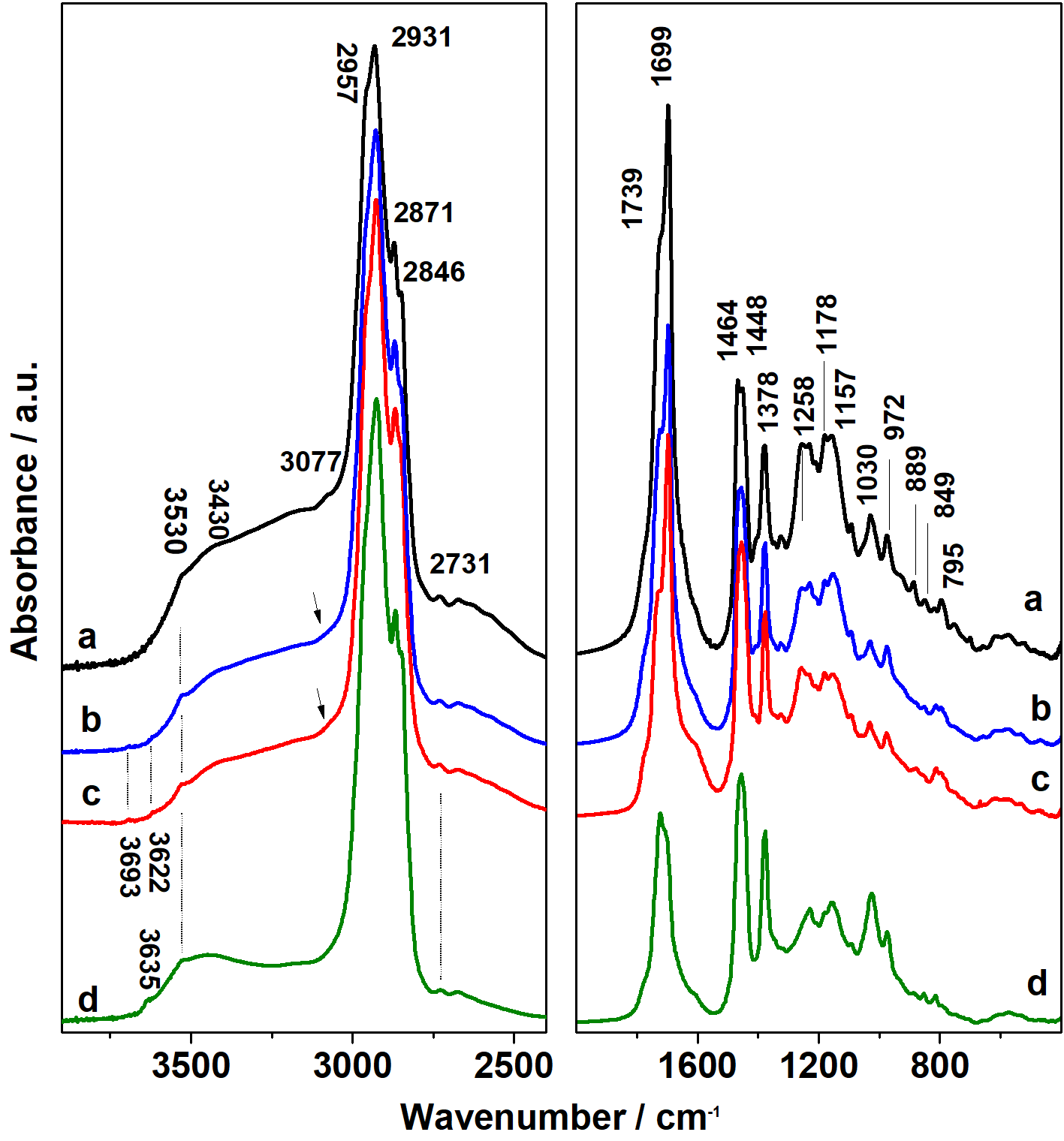


Supplementary Figure 4 IR spectra of Lebanese amber: Aintaourine (a-c), Hammana/ Mdeyrij (d)


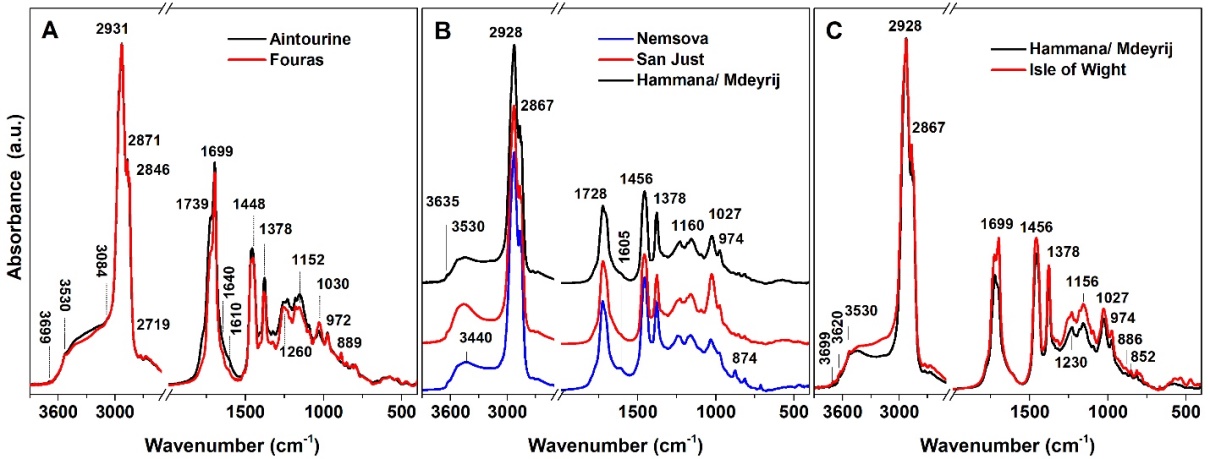


**Supplementary Figure 5** Comparison of IR spectra of Lebanon ambers (Aintourine and Hammana/Mdeyrij) with European ambers Fouras, San Just, Nemšová and Isle of Wight.


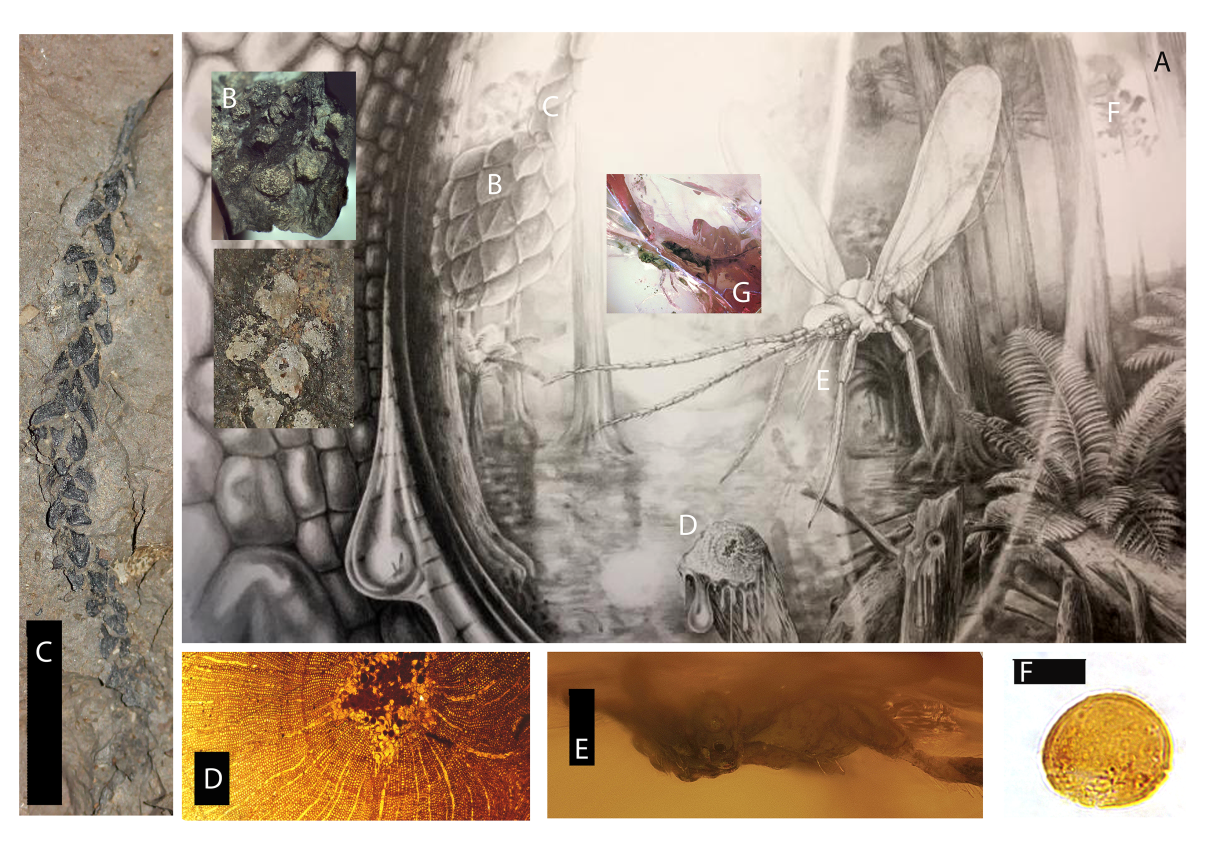


Supplementary Figure 6 The newly described arthropod, *Jankotejacoccus libanogloria* gen. et sp. n., (holotype SNM Z 40023) with a paleoenvironmental reconstruction based on Kimmeridgian fossils discovered in Lebanon from cheirolepidiacean (leave axes, C; wood, D; pollen, F; cone. It represents the earliest scale insect. Scale bars are 1000, 100, 10 μm.

.


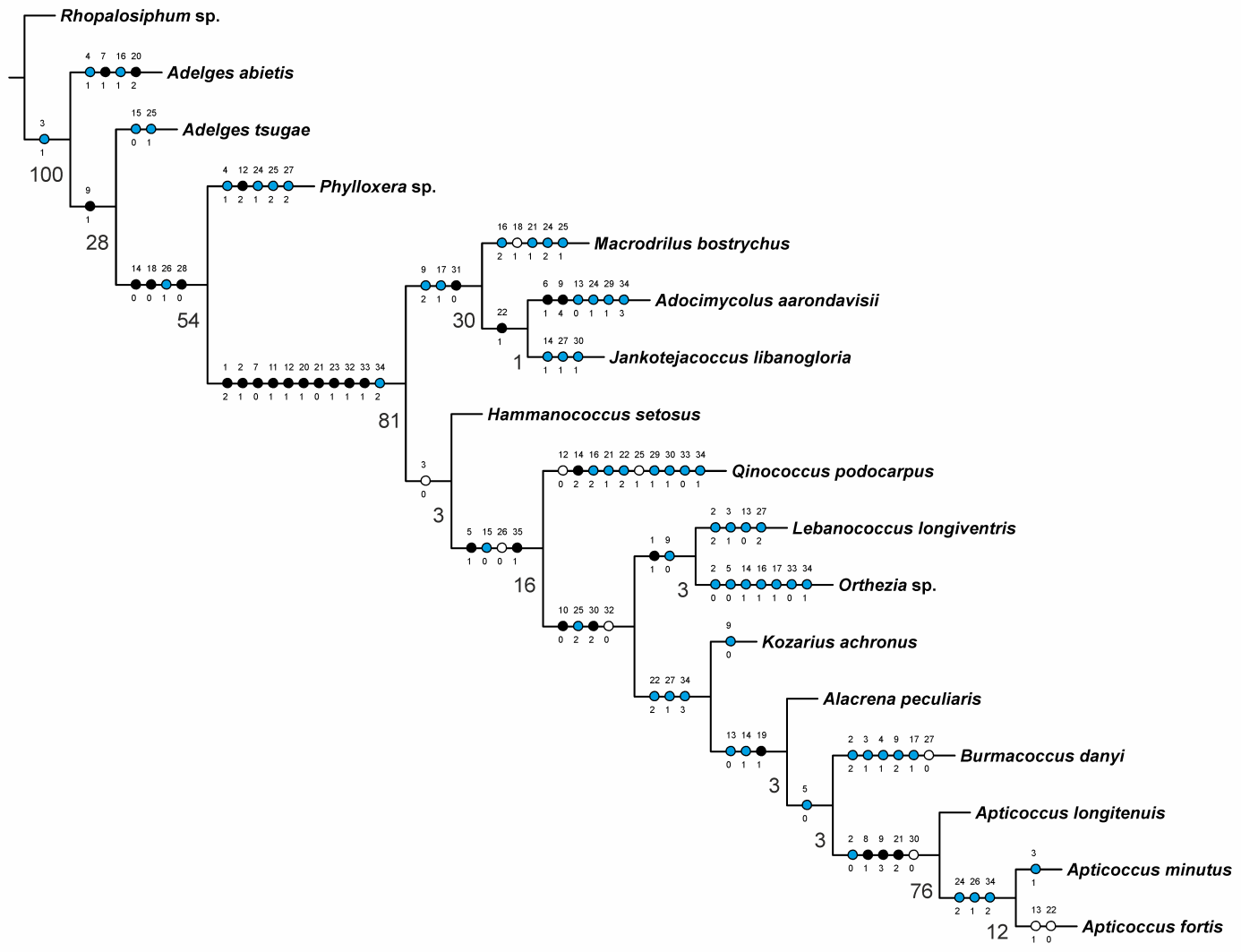


Supplementary Figure 7 ACCTRAN and DELTRAN (Supplementary figure 8) trees with homoplasies marked in blue, apomorphies in black and plesiomorphies in white (as inferred from character distribution analysis). It is topology of NewTechnologySearch Implied Weighting with k=12, single tree, tree 103 steps long and with CI = 52 Ri = 63, bootstrap values are in nodes. Topology and statistics of TS IW k=12 is the same. MR consensus trees of TS and NTS analyses with Equal Weighting are the same, but longer with lower Ci and Ri.

**
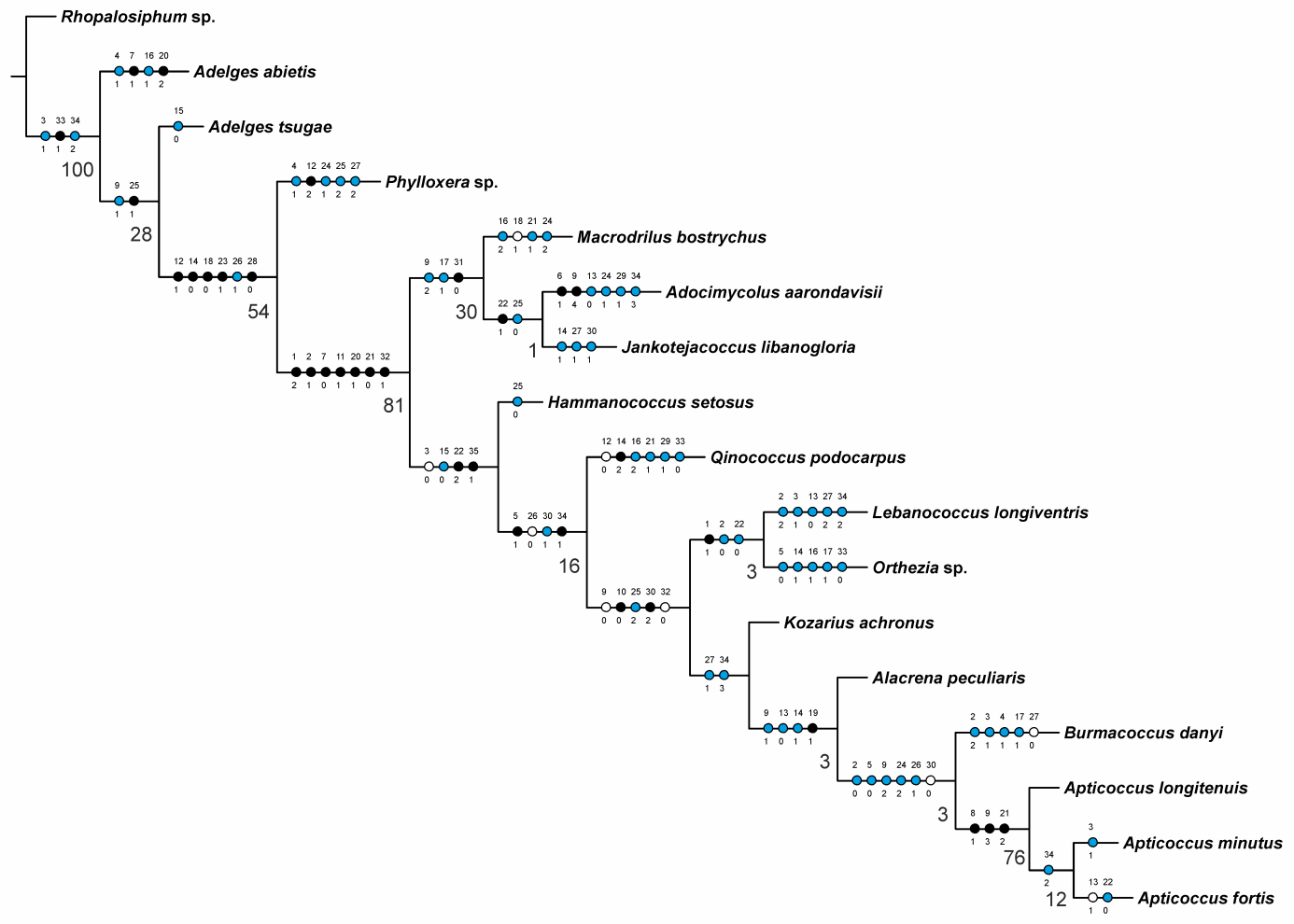
Supplementary Figure 8 The results of parsimony analyses can be interpreted if the analysed taxa come from the same time horizon. Fossil forms, extinct taxa can be (and usually they are) more specialised, presenting numerous novelties (autapomorphies), which are not present in their recent descendants. The most parsimonious tree received is 103 steps long with consistency index = 52 and retention index = 63. Bootstrap values were also calculated, but these are low in most cases, supporting only a few clades revealed. The new family Jankotejacoccidae fam. n. appeared in the clade together with Adocimycolidae and Macrodrilidae, but the support for this clade is very low and relatively high number of homoplastic characters were revealed. Optimizations of character traits under ACCTRAN and DELTRAN were similar, except characters and states 9 (0), 15 (2), 22 (0), 25 (1) and 34 (1) appearing homoplastic in slow optimization (DELTRAN), and 9 (1) homoplastic in fast optimization (ACCTRAN). DELTRAN favours parallelisms over reversals for the same traits.**

**Supplementary Table 1.** Compounds identified in the extract and derivatized extract of SNM Z 40023 AMBER. The most intensive compounds are in boldface type.

**Supplementary Table 2.** Compounds identified in the pyrograms of non-derivatised and derivatised of SNM Z 40023 AMBER. The most intensive compounds are in boldface type.

**Supplementary Table 3.** Reflectance of amber and macerals in amber-bearing sediment.

**Supplementary Table 4** EPMA chemical composition of diaspore/böhmite (in wt.%; diaspore unconfirmed with XRD) and formula calculation (in atoms per formula unit). Contents of V, Cr, Mn, Ni, Zn, Mg, Ca, Na, K, F and Cl are under detection limit. Low analytical totals are due micro/nanoporous material and/or its partial hydratation.

|  | |  |  |
| --- | --- | --- | --- |
|  |  | |  |
| Anal. # | V-1.5 | | V-1.6 |
| SiO_2_ | 0,28 | | 0,05 |
| TiO_2_ | 0,21 | | 0,04 |
| Al_2_O_3_ | 82,19 | | 80,77 |
| Fe_2_O_3_ | 0,33 | | 0,26 |
| H_2_O calc. | 14,65 | | 14,32 |
| Total | 97,66 | | 95,44 |
|  |  | |  |
| Mineral formulae based on | | | |
| cation sum = 1 and (OH) = 1 | | | |
|  |  | |  |
| Si | 0,003 | | 0,001 |
| Ti | 0,002 | | 0,000 |
| Al | 0,991 | | 0,997 |
| Fe | 0,003 | | 0,002 |
| Sum cat. | 0,999 | | 1,000 |
|  |  | |  |
| O | 1,000 | | 1,000 |
| OH | 1,000 | | 1,000 |
| Sum an. | 2,000 | | 2,000 |

**Supplementary Table 5** EPMA chemical composition of hydroxylian pseudorutile (in wt.%) and formula calculation (in atoms per formula unit). Contents of Cr, Ni, Zn, Na, K, F , and Cl are under detection limit. Low analytical totals are due micro/nanoporous material and its partial hydratation.

|  |  |  |  |  |
| --- | --- | --- | --- | --- |
|  |  |  |  |  |
| Anal. # | V-1.1 | V-1.2 | V-1.3 | V-1.4 |
| SiO_2_ | 0,14 | 0,13 | 0,27 | 0,19 |
| TiO_2_ | 62,45 | 61,77 | 60,84 | 61,34 |
| Al_2_O_3_ | 0,41 | 0,25 | 0,90 | 0,68 |
| V_2_O_3_ | 0,41 | 0,25 | 0,38 | 0,37 |
| Fe_2_O_3_ | 27,96 | 28,81 | 27,76 | 27,88 |
| MnO | 0,60 | 0,56 | 0,39 | 0,41 |
| MgO | 1,24 | 1,24 | 1,51 | 1,52 |
| CaO | 0,08 | 0,10 | 0,09 | 0,09 |
| Total | 93,29 | 93,11 | 92,14 | 92,48 |
|  |  |  |  |  |
| Mineral formulae based on Ti = 3 atoms | | | |  |
|  |  |  |  |  |
| Si | 0,009 | 0,008 | 0,018 | 0,012 |
| Ti | 3,000 | 3,000 | 3,000 | 3,000 |
| Al | 0,031 | 0,019 | 0,070 | 0,052 |
| V | 0,021 | 0,013 | 0,020 | 0,019 |
| Fe | 1,344 | 1,400 | 1,370 | 1,364 |
| Mn | 0,032 | 0,031 | 0,022 | 0,023 |
| Mg | 0,118 | 0,119 | 0,148 | 0,147 |
| Ca | 0,005 | 0,007 | 0,006 | 0,006 |
| Sum cat. | 4,560 | 4,597 | 4,654 | 4,623 |
|  |  |  |  |  |
| OH | 1,464 | 1,356 | 1,200 | 1,290 |
| O | 7,536 | 7,644 | 7,800 | 7,710 |
| Sum an. | 9,000 | 9,000 | 9,000 | 9,000 |
|  |  |  |  |  |
| Ti/(Ti+Fe) | 0,691 | 0,682 | 0,686 | 0,687 |

**Supplementary Table 6** Palynological analysis

**Supplementary Table 7** Amber outcrops

**Supplementary Table 8** Character distribution

|  | 1 | 2 | 3 | 4 | 5 | 6 | 7 | 8 | 9 | 10 | 11 | 12 | 13 | 14 | 15 | 16 | 17 | 18 | 19 | 20 | 2 | 22 | 23 | 24 | 25 | 26 | 27 | 28 | 29 | 30 | 31 | 32 | 33 | 34 | 35 |
| --- | --- | --- | --- | --- | --- | --- | --- | --- | --- | --- | --- | --- | --- | --- | --- | --- | --- | --- | --- | --- | --- | --- | --- | --- | --- | --- | --- | --- | --- | --- | --- | --- | --- | --- | --- |
| *Rhopalosiphum* sp. | 0 | 2 | 0 | 0 | 0 | 0 | 2 | 0 | 0 | 1 | 0 | 0 | 1 | 1 | 1 | 0 | 0 | 1 | 0 | 0 | 3 | 0 | 0 | 0 | 0 | 0 | 0 | 1 | 0 | 0 | 1 | 0 | 0 | 0 | 0 |
| *Adelges abietis* | 0 | 2 | 1 | 1 | 0 | 0 | 1 | 0 | 0 | 1 | 0 | 0 | 1 | 1 | 1 | 1 | 0 | 1 | 0 | 2 | 3 | 0 | 0 | 0 | 0 | 0 | 0 | 1 | 0 | - | 1 | 0 | ? | ? | ? |
| *Adelges tsugae* | 0 | 2 | 1 | 0 | 0 | 0 | 2 | 0 | 1 | 1 | 0 | 0 | 1 | 1 | 0 | 0 | 0 | 1 | 0 | 0 | 3 | 0 | 0 | 0 | 1 | 0 | 0 | 1 | 0 | - | 1 | 0 | ? | ? | ? |
| *Phylloxera* sp. | 0 | 2 | 1 | 1 | 0 | 0 | 2 | 0 | 1 | 1 | 0 | 2 | 1 | 0 | 1 | 0 | 0 | 0 | 0 | 0 | 3 | 0 | - | 1 | 2 | 1 | 2 | 0 | 0 | - | 1 | 0 | ? | ? | ? |
| *Adocimycolus aarondavisii* | 2 | 1 | 1 | 0 | 0 | 1 | 0 | 0 | 4 | 1 | 1 | 1 | 0 | 0 | 1 | 0 | 1 | 0 | 0 | 1 | 0 | 1 | 1 | 1 | 0 | 1 | 0 | 0 | 1 | 0 | 0 | 1 | 1 | 3 | ? |
| *Macrodrilus bostrychus* | 2 | 1 | 1 | 0 | 0 | 0 | 0 | 0 | 2 | 1 | 1 | 1 | 1 | 0 | 1 | 2 | 1 | 1 | 0 | 1 | 1 | 0 | - | 2 | 1 | 1 | 0 | 0 | 0 | 0 | 0 | 1 | ? | ? | 0 |
| *Jankotejacoccus libenoglori* | 2 | 1 | 1 | 0 | 0 | 0 | 0 | 0 | 2 | ? | ? | ? | 1 | 1 | 1 | 0 | 1 | 0 | 0 | 1 | 0 | 1 | 1 | 0 | 0 | 1 | 1 | 0 | 0 | 1 | ? | 1 | 1 | 2 | ? |
| *Apticoccus minutus* | 2 | 0 | 1 | 0 | 0 | 0 | 0 | 1 | 3 | 0 | 1 | 1 | 0 | 1 | 0 | 0 | 0 | 0 | 1 | 1 | 2 | 2 | 1 | 2 | 2 | 1 | 1 | 0 | 0 | 0 | 1 | 0 | 1 | 2 | 1 |
| *Apticoccus longitenuis* | 2 | 0 | 0 | 0 | 0 | 0 | ? | 1 | 3 | 0 | 1 | 1 | 0 | 1 | 0 | 0 | 0 | 0 | 1 | 1 | 2 | 2 | 1 | ? | 2 | ? | 1 | 0 | 0 | 0 | 1 | 0 | 1 | 3 | 1 |
| *Apticoccus fortis* | 2 | 0 | 0 | 0 | 0 | 0 | ? | 1 | 3 | 0 | 1 | 1 | 1 | 1 | 0 | 0 | 0 | 0 | 1 | 1 | 2 | 0 | 1 | 2 | 2 | 1 | 1 | 0 | 0 | - | 1 | 0 | 1 | 2 | 1 |
| *Alacrena peculiaris* | 2 | 1 | 0 | 0 | 1 | 0 | 0 | 0 | 1 | 0 | 1 | 1 | 0 | 1 | 0 | 0 | 0 | 0 | 1 | 1 | 0 | 2 | 1 | 0 | 2 | 0 | 1 | 0 | 0 | 2 | 1 | 0 | 1 | 3 | ? |
| *Kozarius achronus* | 2 | 1 | 0 | 0 | 1 | 0 | 0 | 0 | 0 | 0 | 1 | 1 | 1 | 0 | 0 | 0 | 0 | 0 | 0 | 1 | 0 | 2 | 1 | 0 | 2 | 0 | 1 | 0 | 0 | 2 | 1 | 0 | 1 | 3 | 1 |
| *Burmacoccus danyi* | 2 | 2 | 1 | 1 | 0 | 0 | 0 | 0 | 2 | 0 | 1 | 1 | 0 | 1 | 0 | 0 | 1 | 0 | 1 | 1 | 0 | 2 | 1 | ? | 2 | ? | 0 | 0 | 0 | ? | 1 | 0 | 1 | 3 | ? |
| *Lebanococcus longiventris* | 1 | 2 | 1 | 0 | 1 | 0 | 0 | 0 | 0 | 0 | 1 | 1 | 0 | 0 | 0 | 0 | 0 | 0 | 0 | 1 | 0 | 0 | 1 | 0 | 2 | 0 | 2 | 0 | 0 | 2 | 1 | 0 | 1 | 2 | ? |
| *Hammanococcus setosus* | 2 | 1 | 0 | 0 | 0 | 0 | 0 | 0 | 1 | 1 | 1 | 1 | ? | ? | ? | ? | 0 | ? | - | 1 | ? | - | - | 0 | 0 | 1 | 0 | 0 | 0 | 0 | 1 | 1 | 1 | 2 | ? |
| *Orthezia* sp. | 1 | 0 | 0 | 0 | 0 | 0 | 0 | 0 | 0 | 0 | 1 | 1 | 1 | 1 | 0 | 1 | 1 | 0 | 0 | 1 | 0 | 0 | 1 | 0 | 2 | 0 | 0 | 0 | 0 | 2 | 1 | 0 | 0 | 1 | 1 |
| *Qinococcus podocarpus* | 2 | 1 | 0 | 0 | 1 | 0 | 0 | 0 | 1 | 1 | 1 | 0 | 1 | 2 | 0 | 2 | 0 | 0 | 0 | 1 | 1 | 2 | 1 | 0 | 1 | 0 | 0 | 0 | 1 | 1 | 1 | 1 | 0 | 1 | 1 |

**Supplementary references**

1. Sendi H, Hinkelman J and Vršanská L *et al.* Roach nectarivory, gymnosperm and earliest flower pollination evidence from Cretaceous ambers. *Biologia* 2020; **75**: 1613–1630.

2. Bray PS and Anderson KB. The nature and fate of natural resins in the geosphere XIII: a probable pinaceous resin from the early Cretaceous (Barremian), Isle of Wight. *Geochem T* 2008; **9**: 3.

3. Peñalver E, Delclós X and Soriano C. A new rich amber outcrop with palaeobiological inclusions in the Lower Cretaceous of Spain. *Cretaceous Res* 2007; **28**: 791–802.

4. Moreau JD, Néraudeau D and Perrichot V. Conifers from the Cenomanian amber of Fouras (Charente-Maritime, western France). *BSGF - Earth Sci Bull* 2020; **191**: 16.

**5. Linnaeus C. *Systema naturae per regna tria naturae, secundum classes, ordines, genera, species, cum characteribus, differentiis, synonymis, locis.* Tomus I. Editio decima, reformata. Laurentii Salvii, Holmiæs, 1758.**

**6. Amyot CJ and Audinet-Serville JG. *Deuxième partie. Homoptères. Homoptera Latr. Histoire naturelle des insectes. Hemiptères*. Librairie encyclopédique de Roret, 1843.**

**7. Fallén CF. *Specimen novam Hemiptera disponendi methodum exhibiens.* Lundae, 1814.**

**8. Heslop-Harrison G. Prelimary notes on the ancestry, family relations, evolution and speciation of the homopterous Psyllidae. – II. *Ann Mag Nat* 1952; 5(12): 679–696.**

**9. Borchsenius NS. *Chervetsy i shchitovki SSSR (Coccoidea)*. Akademia Nauk SSSR, 1950.**

**10. Bodenheimer FS. The Coccoidea of Turkey. I. *Revue de la Faculté des Sciences de l’Université d’Istanbul* (Sér. B). 1952; 17: 315–351.**

**11. Koteja J. On the phylogeny and classification of the scale insects (Homoptera, Coccinea) (discussion based on the morphology of the mouthparts). *Acta zoologica cracoviensia* 1974; 14: 267–325.**

**12.** García Morales M, Denno BD and Miller DR *et al.* ScaleNet: a literature-based model of scale insect biology and systematics. *Database,* 2016: bav118.

**13.** Ross L, Pen I and Shuker DM. Genomic conflict in scale insects: the causes and consequences of bizarre genetic systems. *Biol Rev* 2010; **85:** 807–828.

**14. Hodgson CJ and Hardy NB. The phylogeny of the superfamily Coccoidea (Hemiptera: Sternorrhyncha) based on the morphology of extant and extinct macropterous males. *Syst Entomol* 2013; 38:794–804.**

**15. Wang B, Xia F and Wappler** *et al.* **Brood care in a 100-million-year-old scale insect. *Elive* 2015; 4, e05447.**

**16. Hardy NB.** The Biodiversity of Sternorrhyncha: Scale Insects, Aphids, Psyllids, and Whiteflies. **In: Foottit AG and Adler PH (Eds). Insect biodiversity: Science and society, Wiley, Chichester, 2018, 591–625.**

17. Drohojowska J, Szwedo J and Žyla D *et al.* Fossils reshape the Sternorrhyncha evolutionary tree (Insecta, Hemiptera). *Sci Rep-UK* 2020; **10**: 11390.

18. Vea IM and Grimaldi DA. Putting scales into evolutionary time: the divergence of major scale insect lineages (Hemiptera) predates the radiation of modern angiosperm hosts. *Sci Rep-UK* 2016; **6**: 23487.

**19. Koteja J. Essay on the prehistory of the scale insects (Homoptera, Coccinea). *Ann Zool* 1985; 38(15): 461–503.**

**20. Kozár F. *Ortheziidae of the World.*** Hungarian Academy of Sciences, **Budapest, Hungary**: Plant Protection Institute, **2004.**

**21. Foldi I. Ground pearls: a generic revision of the Margarodidae *sensu stricto* (Hemiptera: Sternorrhyncha: Coccoidea). *Ann Soc Entomol Fr* 2005; 41(1): 81–125.**

**22. Vahedi HA and Hodgson CJ. Illustrated monograph of scale Insects: Hypogaeic margarodids and a discussion on their phylogenetic relationship. Chisinau: LAP, 2012.**

**23. Tang F. *The Pseudococcidae of China (Homoptera: Coccoidea of Insecta*).** Chinese Agricultural Science Technology Press, **1992.**

**24.** Downie DA and Gullan PJ. Phylogenetic analysis of mealybugs (Hemiptera: Coccoidea: Pseudococcidae) based on DNA sequences from three nuclear genes, and a review of the higher classification. *Syst Entomol* 2004; **29**: 238–260.

**25. Hodgson CJ. In: Ben-Dov Y, Hodgson CJ (eds.). *Soft scale insects - their biology, natural enemies and control*. Elsevier, Amsterdam, 1997: 157–201.**

**26. Hodgson CJ. Comparison of the morphology of the adult males of the rhizoecine, phenacoccine and pseudococcine mealybugs (Hemiptera: Sternorrhyncha: Coccoidea), with the recognition of the family Rhizoecidae Williams. *Zootaxa* 2012; 3291: 1–79.**

**27. Hodgson CJ and Henderson RC. Coccidae (Insecta: Hemiptera: Cooccoidea). *Fauna N Z* 2000; 41: 1–264.**

**28. Koteja J. Paleontology. In: D. Rosen (ed.). *World Crop Pests, Armored Scale Insects: Their Biology, Natural Enemies and Control.* Elsevier, Amsterdam, 1990, 149–163.**

**29. Koteja J. Scale insects (Homoptera, Coccinea) from Upper Cretaceous New Jersey amber. In: Grimaldi DA (ed.) *Studies on fossils in amber, with particular reference to the Cretaceous of New Jersey.* Backhuys Publishers, 2000a, 147–229.**

**30. Koteja J. Advances in the study of fossil coccids (Hemiptera: Coccinea). *Pol Pis Entomol* 2000b; 69: 187–218.**

**31. Koteja J. Xylococcidae and related groups (Hemiptera: Coccinea) from Baltic amber. *Prace Muzeum Ziemi* 2008; 49: 19–56.**

**32. Koteja J and Azar D. Scale insects from Lower Cretaceous amber of Lebanon (Hemiptera: Sternorrhyncha: Coccinea). *Alavesia* 2008; 2: 133–167.**

**33. Anderson J, Anderson H and Cleal C. *Brief history of the gymnosperms: classification, biodiversity, phytogeography and ecology.* SANBI, Pretoria, 2007.**

**34. Taylor EL, Taylor TN and Krings M. *Paleobotany: the biology and evolution of fossil plants*. AP, Cambridge, 2009.**

**35.** Condamine FL, Silvestro D and Koppelhus EB *et al.* The rise of angiosperms pushed conifers to decline during global cooling. *PNAS* 2020; **117**(46): 28867–28875.

**36. Silvestro D, Cascales-Miñana B., C. D., Bacon, A. Antonelli, Revisiting the origin and diversification of vascular plants through a comprehensive Bayesian analysis of the fossil record. *New Phytol* 2015; 207(2): 425–436.**

**37.** Condamine FL, Clapham ME and Kergoat GJ. Global patterns of insect diversification: Towards a reconciliation of fossil and molecular evidence? *Sci Rep* 2016; **6**: 19208.

**38. Krzemiñski W and Krzeminska E. Triassic Diptera: desriptions, revisions and phylogenetic relations. *Acta Zool Cracov* 2003; 46: 153–184.**

**39. Béthoux O, Papier F and Nel A. The Triassic radiation of the entomofauna. *C R Palevol* 2005; 4(6-7): 609–621.**

**40. Blagoderov VA, Grimaldi D, and Fraser NC. How time flies for flies: diverse Diptera from the Triassic of Virginia and early radiation of the order. *Am Mus Novi* 2007; 3572: 1–39.**

**41.** Lukashevich ED, Przhiboro AA and Marchal-Papier F. The oldest occurrence of immature Diptera (Insecta), Middle Triassic, France. *Ann Soc Entomol Fr* 2010; **46**(1-2): 4–22.

**42. Szwedo J. The unity, diversity and conformity of bugs (Hemiptera) through time. *Earth Env Sci T R So.* 2018; 107: 109–128.**

**43. Schuh RT and Weirauch C. *True bugs of the World (Hemiptera: Heteroptera): classification and natural history*. SSP, Manchester, 2020.**

**44.** Fikáček M, Beutel RG and Cai C *et al.* Reliable placement of beetle fossils via phylogenetic analyses – Triassic Leehermania as a case study (Staphylinidae or Myxophaga?). *Syst Entomol* 2020; **45**: 175–187.

**45. van Eldijk TJB, Wappler T and Strother PK** *et al.* **A Triassic-Jurassic window into the evolution of Lepidoptera. *Sci Adv* 2018; 4(1): e1701568.**

**46. Zheng D, Chang S, Wang H** *et al.* **Middle-Late Triassic insect radiation revealed by diverse fossils and isotopic ages from China. *Sci Adv* 2018; 4(9): eaat1380.**

**47. Tihelka E, Cai C and Giacomelli MD** *et al.* **Integrated phylogenomic and fossil evidence of stick and leaf insects (Phasmatodea) reveal a Permian–Triassic co-origination with insectivores. *Roy Soc Open Sci* 2020; 7(11): 7201689.**

**48. Corso JD, Bernardi M and Sun Y** *et al.* **Extinction and dawn of the modern world in the Carnian (Late Triassic). *Sci Adv* 2020; 6(38): eaba0099.**

**49.** Ruffell A, Simms MJ and Wignall PB. The Carnian Humid Episode of the late Triassic: A review. *Geol Mag* 2016; **153**: 271–284.

**50. Miller CS, Peterse F and da Silva AC** *et al.* **Astronomical age constraints and extinction mechanisms of the Late Triassic Carnian crisis. *Sci Rep-Uk* 2017; 7: 2557.**

**51. Miller CS and Baranyi V. Triassic Climates. In: Alderton D and Scott SA. Elias AP (eds.). *Encyclopedia of Geology (Second Edition)*, Cambridge, Massachusetts, 2021, 514–524.**

**52. Roghi G, Ragazzi E and Gianolla P. Triassic Amber of the Southern Alps (Italy). *Palaios* 2006; 21(2): 143–154.**

**53.** Schmidt AR, Jancke S and Lindquist EE *et al***.** Arthropods in amber from the Triassic Period. *Natl Acad Sci* 2012; **109(37): 14796–14801.**

**54. Stilwell J, Langendam A and Mays C** *et al***. Amber from the Triassic to Paleogene of Australia and New Zealand as exceptional preservation of poorly known terrestrial ecosystems. *Sci Rep* 2020; 10: 5703.**

**55. Furin S, Preto N and Rigo M** *et al*. **Bowring, High-precision U-Pb zircon age from the Triassic of Italy: Implications for the Triassic time scale and the Carnian origin of calcareous nannoplankton and dinosaurs. *Geology* 2006; 34 (12): 1009–1012.**

**56. Dal Corso J, Mietto P and Newton RJ** *et al*. **Discovery of a major negative δ^13^C spike in the Carnian (Late Triassic) linked to the eruption of Wrangellia flood basalts. *Geology* 2012; 40(1): 79–82.**

**57. Fraser N and Sues H. The beginning of the ‘Age of Dinosaurs’: A brief overview of terrestrial biotic changes during the Triassic. *Earth Env Sci T R SO.*2011; 101(3-4): 189–200.**

**58.** Jones ME, Anderson CL and Hipsley CA *et al*. Integration of molecules and new fossils supports a Triassic origin for Lepidosauria (lizards, snakes, and tuatara). *BMC Evol Biol* 2013; **13**: 208.

**59.** Bernardi M, Gianolla P and Petti FM *et al*. Dinosaur diversification linked with the Carnian Pluvial Episode. *Nat Commun* 2018; **9**: 1-10.

**60. Shcherbakov DE. Extinct four–winged ancestors of scale insects (Homoptera: Sternorrhyncha). In: *Proceedings of the Sixth International Symposium of scale insect Studies, part II, Cracow, 1990*. Agricultural University Press, Kraków, Poland.**

**61. Dziedzicka A. Badania porównawcze nad odnóżami czerwców (Coccinea). *Prace monograficzne Wyższej Szkoły Pedagogicznej w Krakowie.* 1977; 20: 1–107.**

62. Giliomee JH. The Adult Male. In: Ben-Dov Y, Hodgson CJ (eds.). *Soft Scale Insects. Their Biology, Natural Enemies and Control*, Elsevier, 1997, 23-30.

**63.** Dunn JA. Antennal sensilla of vegetalble aphids. *Entomol Exp Appl* 1978; **24**(3): 148–149.

**64. Shaposhnikov GK. Oligomerizatsiya, polymerizatsiya i uporyadochenie morfologicheskikh struktur v evolyutsii tley (Homoptera, Aphidinea). *Entomologicheskoe Obozrenie* 1979; 58(4): 716–741.**

**65. Sullivan DJ. Aphids (Hemiptera: Aphididae). In: Capinera, JL (ed.), *Encyclopedia of Entomology.* Dordrecht: Springer, 2008, 107–110.**

**66. Fink R. Morphologische und physiologische untersuchungen an den intrazellularen symbionten von *Pseudococcus citri*. *Zeitschrift für Morphologie und Ökologie der Tiere* 1952; 41(1): 78–146.**

**67. Buchner P. *Endosymbiosis of animals with plant microorganisms.*New York: Interscience Publishers, 1965.**

**68. Buchner P. Endosymbiosestudien an Schildläusen. VIII. Die Symbiosen der Paleococcoidea. *Zeitschrift für Morphologie und Ökologie der Tiere* 1966; 56(1): 275–362.**

**69.** Rosenblueth M, Martínez-Romero J and Tabita Ramírez-Puebla S *et al*. Endosymbiotic microorganisms of scale insects. *TIP Rev Esp Cienc Quím Biol* 2018; **21**: 53–69.

**70. Baumann P, Moran N and Baumann L** *et al*. **The prokaryotes. In: Baumann P, Moran N and Baumann L. *The prokaryotes.* New York: Springer, 2000, 155-189.**

**71.** Ross L, Shuker DM and Normark BB *et al*. The role of endosymbionts in the evolution of haploid-male genetic systems in scale insects (Coccoidea). *Ecol Evol* 2012; **2**(5): 1071–1081

72**.** Michalik K, Szklarzewicz T and Kalandyk-Kołodziejczyk *et al*. Bacterial associates of Orthezia urticae, Matsucoccus pini, and Steingelia gorodetskia - scale insects of archaeoccoid families Ortheziidae, Matsucoccidae, and Steingeliidae (Hemiptera, Coccomorpha). *Protoplasma* 2019; **256**(5): 1205–1215.

73**.** Otto A and Wilde V. Sesqui-, Di-, and triterpenoids as chemosystematic markers in extant conifers - *A review Bot Rev* 2001; **67**: 141–238.

74. Anderson KB. The nature and fate of natural resins in the geosphere. XII. Investigation of C-ring aromatic diterpenoids in Raritan amber by pyrolysis-GC-matrix isolation FTIR-MS. *Geochem T* 2006: **7**.

**75. Anderson KB, Winans RE and Botto RE. The nature and fate of natural resins in the geosphere II. Identification, classification and nomenclature of resinites. *Org Geoch* 1992; 18(6): 829-841.**

76. Menor-Salván C, Najarro M and Velasco F *et al*. Terpenoids in extracts of lower cretaceous ambers from the Basque-Cantabrian Basin (El Soplao, Cantabria, Spain): paleochemotaxonomic aspects. *Org Geochem* 2010; **41**: 1089–1103.

77. Sýkorová I, Pickel W and Christanis K *et al*. Classification of huminite – ICCP system 1994. *Int J Coal Geol* 2005; **62**: 85–106.

78. Burnham AK and Sweeney JJ. A chemical kinetic model of vitrinite reflectance and 390 maturation. *Geochim Cosmochim Ac* 1989; **53**: 2649–2657.

79. Petersen HI. Morphology, formation and palaeo-environmental implications of naturally formed char particles in coals and carbonaceous mudstones. *Fuel* 1998; **77**(11): 1177–1183.

80. Kwiecińska B and Petersen HJ. Graphite, Semi-graphite, natural coke, and natura char classification - ICCP system. *Int J Coal Geol* 2004; **57**: 99–116.

81. Chen Y, Shah N, Braun A *et al*. Electron Microscopy Investigation of Carbonaceousparticulate matter generated by combustion of fossil fuels. *Energ Fuel* 2005; **19**: 4, 1644–1651.

82. Jones TP, Scott AC and Cope M. Reflectance measurements and the temperature of formation of modern charcoals and implications for studies of fusain. *B Soc Géol Fr* 1991; **162**(2): 193–200.

83. Scott AC, Glasspool IJ. Charcoal reflectance as a proxy for the emplacement temperature of pyroclastic flow deposits. *Geology* 2005; **33**: 589–592.

84. Scott AC and Glasspool IJ. Observations and experiments on the origin and formation of inertinite group macerals. *Int J Coal Geol* 2007; **70**: 55–66.

85. Bunt JR, Joubert JP and Waanders FB. Coal char temperature profile estimation using optical reflectance for a commercial-scale Sasol-Lurgi FBDB gasifier. *Fuel* 2008; **87**: 2849–2855.

86. McParland LC, Collinson ME and Scott AC, G. Campbell, The use of reflectance values for the interpretation of natural and anthropogenic charcoal assemblages. *Archaeol Anthrop Sci* 2009; **1**; 249–261.

87. Petersen HI and Lindström S. Synchronous Wildfire Activity Rise and Mire Deforestation at the Triassic–Jurassic Boundary. *PLOS One* 2012; **7**: e47236.

88. Hudspith VA, Belcher CM and Yearsley JM. Charring temperatures are driven by the fuel types burned in a peatland wildfire. *Front Plant Sci* 2014; **5**(13): 714.

89. Murchison DG. Properties of Coal Macerals. *Coal Sci* 1966; 307–331.

90. Murchison DG. Resinite: its infrared spectrum and coalification pattern. *Fuel* 1976; **55**: 79–83.

91. Murae T, Shimokawa S and Aihara A. Pyrolytic and spectroscopic studies of the diagenetic alteration of resinites. In: *Amber, Resinite, and Fossil Resins*, Anderson, K.B., Crelling, J.C. (Eds.) ACS Symposium Series 1995; **617**: 76–91.

92. Kotulová J, Starek D and Havelcová M *et al.* Amber and organic matter from the late Oligocene deep-water deposits of the Central Western Carpathians (Orava–Podhale Basin). *Int J of Coal Geol* 2019; **207**: 96–109.

93. Pastorelli G. Archaeological Baltic amber: Degradation mechanisms and conservation measures. *Ph.D. Thesis*, University of Bologna, 2009.

94. Shashoua Y, Degn Berthelsen MBL and Nielsen OF. Raman and ATR-FTIR spectroscopies applied to the conservation of archaeological Baltic amber. *J Ram Spectrosc* 2006; **37**(10): 1221–1227.

95. Diessel CFK. An appraisal of lignite facies based on maceral characteristics. *Aust Lignite Geol* 1982; **4**(2): 474–484.

96. Diessel CFK. *Coal-Bearing Depositional Systems.*Berlin-Heidelberg: Springer-Verlag, 1992.

97. Littke R, Sachsenhofer RF. Organic petrology of deep sea sediments: a compilation of results from the Ocean Drilling Program and the Deep Sea Drilling Project. *Energy Fuels* 1994; **8**: 1498–1512.

98. Sun YZ, Lin M and Li H *et al.* Maceral and Geochemical Characteristics of Oil Shale 2 from the Huangxian Basin, China. *Energ Explor Exploit* 2001; **19**(6): 569–580.

99. Poinar Jr GO and Mastalerz M. Taphonomy of fossilized resins: determining the biostratinomy of amber. *Acta Geol Hisp* 2000; **35**: 171–182.

100. Najarro M, Peñalver E and Pérez-de la Fuente R *et al.* Review of the El Soplao amber outcrop, Early Cretaceous of Cantabria, Spain. *Acta Geol Sin* 2010; **84**(4): 959–976.

101. Sykorova V, Dvorakova S and Vcelak J *et al.* Search for new genetic biomarkers in poorly differentiated and anaplastic thyroid carcinomas using next generation sequencing. *Anticancer Res* 2015;**35**(4): 2029–2036.

102. Pickel W, Kus J and Flores D *et al.* Classification of liptinite –ICCP system 1994. *Int J Coal Geol* 2017; **169**: 40–61.

103. Cabrera L, Hagemann HW, Pickel W *et al.* The coal-bearing, Cenozoic As Pontes Basin (northwestern Spain): geological influence on coal characteristics. *Int J Coal Geol* 1995; **27**: 201–226.

104. Berner RA and Raiswell R. C/S method for distinguishing freshwater from marine sedimentary rocks. *Geology* 1984; **12**: 365–368.

105. Beck CW. Spectroscopic Investigations of Amber. *Appl Spectrosc* 1986; **22**: 57-110.

106. Guiliano M, Asia L and Onoratini G *et al.* Applications of diamond crystal ATR FTIR spectroscopy to the characterization of ambers. *Spectrochim. Acta A* 2007; **67**: 1407.

107. Pastorelli G, Shashoua Y and Richter J. Hydrolysis of Baltic amber during thermal ageing – An infrared. *Spectrochim Acta A* 2007; **67**: 1407–1411.

108. Poulin J and Helwig K. The characterisation of amber from deposit sites in western and northern Canada. *J Archaeol Sci* 2016; **7**: 155–168.

109. Mays C, Coward AJ and O'Dell LA *et al.* The botanical provenance and taphonomy of Late Cretaceous Chatham amber, Chatham Islands, New Zealand. *Rev Palaeobot Palyno* 2019; **260**: 16–26.

110. Pagacz J, Naglik B and Stach P *et al.* Maturation process of natural resins recorded in their thermal properties. *J Mater Sci* 2020; **55**: 4504–4523.

111. Madejova J, Kečkéš J and Pálková H *et al.* Identification of components in smectite/kaolinite mixtures. *Clay Miner* 2001; **37**: 377–388.

112. Farmer VC. Infrared Spectra of Minerals. London, UK: Mineralogical Society, 1974.

113. Havelcová M, Machovič V, Špaldoňová A *et al.* Characterization of Eocene fossil resin from Moravia, Czech Republic: Insights into macromolecular structure. *Spectrochim. Acta A: Molecular Spectroscopy* 2019; **215**: 176–186.

114. Brasier AT, McIlroy D, McLoughlin N. *Earth System Evolution and Early Life: A Celebration of the Work of Martin Brasier* 44. London: Geological Society of London, 2017.

115. Coward AJ, Mays C and Patti AF *et al.* Taphonomy and chemotaxonomy of Eocene amber from southeastern Australia. *Org Geochem* 2018; **118:** 103–115.

116. Nohra Y, Azar D and Gèze R *et al.* New Jurassic amber outcrops from Lebanon. *Terr Arthropod Rev* 2013; **6**: 27–51.

117. Nohra Y, Perrichot V and Jeanneau L *et al.* Chemical Characterization and Botanical Origin of French Ambers. *J Nat Prod* 2015; **78**: 1284–1293.

118. Nicholas CJ, Henwood AA and Simpson M. A new discovery of early Cretaceous(Wealden) amber from the Isle of Wight. *Geol Mag* 1993; **130**: 847–85.

**119. Maddison WP and Maddison DR. Mesquite: a modular system for evolutionary analysis. Version 3.81; 2023.** <http://www.mesquiteproject.org>**.**

**120. Goloboff PA and Morales ME. TNT version 1.6, with a graphical interface for MacOS and Linux, including new routines in parallel. *Cladistics* 2023; 39(2): 144–153.**

**121. Nixon KC. ASADO, version 1.85 TNT-MrBayes Slaver version 2; mxram 200 (vl. 5.30). Published by the author, Ithaca, New York, 2008.**

122 Congreve CR and Lamsdell JC Implied weighting and its utility in palaeontological datasets: a study using modelled phylogenetic matrices. *Palaeontol* 2016; **59**(3): 447–465.

123. Goloboff PA, Torres A and Arias JS. Weighted parsimony outperforms other methods of phylogenetic inference under models appropriate for morphology. *Cladistics* 2018; **34**(4), 407–437.

124. **Agnarsson I and Miller JA. Is ACCTRAN better than DELTRAN? *Cladistics* 2008; 24 (6), 1032–1038.**
